# Enhanced Photodynamic Cancer Treatment by Mitochondria‐Targeting and Brominated Near‐Infrared Fluorophores

**DOI:** 10.1002/advs.201700481

**Published:** 2017-12-19

**Authors:** Ilkoo Noh, DaeYong Lee, Heegon Kim, Chan‐Uk Jeong, Yunsoo Lee, Jung‐Oh Ahn, Hoon Hyun, Ji‐Ho Park, Yeu‐Chun Kim

**Affiliations:** ^1^ Department of Chemical and Biomolecular Engineering Korea Advanced Institute of Science and Technology (KAIST) Daejeon 305‐701 South Korea; ^2^ Department of Bio and Brain Engineering Korea Advanced Institute of Science and Technology (KAIST) Daejeon 305‐701 South Korea; ^3^ Korea Research Institute of Bioscience and Biotechnology 52 Eoeun‐dong Daejon 305‐333 South Korea; ^4^ Department of Biomedical Sciences Chonnam National University Medical School Gwangju 501‐746 South Korea

**Keywords:** cancer therapy, heptamethine cyanine dye, mitochondria targeting, near‐infrared (NIR) dye, photodynamic therapy

## Abstract

A noninvasive and selective therapy, photodynamic therapy (PDT) is widely researched in clinical fields; however, the lower efficiency of PDT can induce unexpected side effects. Mitochondria are extensively researched as target sites to maximize PDT effects because they play crucial roles in metabolism and can be used as cancer markers due to their high transmembrane potential. Here, a mitochondria targeting photodynamic therapeutic agent (MitDt) is developed. This photosensitizer is synthesized from heptamethine cyanine dyes, which are conjugated or modified as follows. The heptamethine meso‐position is conjugated with a triphenylphosphonium derivative for mitochondrial targeting, the *N*‐alkyl side chain is modified for regulation of charge balance and solubility, and the indolenine groups are brominated to enhance reactive oxygen species generation (ROS) after laser irradiation. The synthesized MitDt increases the cancer uptake efficiency due to the lipo‐cationic properties of the triphenylphosphonium, and the PDT effects of MitDt are amplified after laser irradiation because mitochondria are susceptible to ROS, the response to which triggers an apoptotic anticancer effect. Consequently, these hypotheses are demonstrated by in vitro and in vivo studies, and the results indicate strong potential for use of MitDts as efficient single‐molecule‐based PDT agents for cancer treatment.

## 1 Introduction

Phototherapy methods, including photodynamic therapy (PDT), have been intensively studied in clinical fields as a method to solve drug resistance, frequent relapses, and toxicity, which are problems associated with the use of conventional therapeutic agents.^1^, ^2^ PDT is a process by which a photosensitizer (PS) absorbs light energy and then converts oxygen to singlet oxygen or free radicals, resulting in programmed cell death.^1^ The therapeutic effect of PDT is achieved by the formation of singlet oxygen and free radicals after laser irradiation; it thus has characteristics other than those of other therapeutic agents. The advantages of PDT are that it is a noninvasive treatment and the generated reactive oxygen species (ROS) have a short lifetime and diffusion range (0.1 µm); treatment at restricted sites is therefore possible.^2^, ^3^ However, PDT also has some drawbacks as a first‐line therapy because its effectiveness is reduced when the oxygen supply is lowered in tumors due to their microvessel structure and microcirculation, and because of oxygen consumption by the PDT process.^4^ In addition, PDT agents may cause genetic variation when they are taken up by organelles such as the nucleus.^5^ In order to overcome these drawbacks, research has been conducted to develop a PS target site where there is a mechanism for action by PDT.^6^

Among the organelles of mammalian cells, mitochondria are vital subcellular organelles and play crucial roles in metabolism including cell growth, and their actions are intricately interwoven into signal transduction and the apoptotic process.^7^, ^8^ In addition, the transmembrane potential of mitochondria is negative internally. This internal charge attracts cationic agents (e.g., lipo‐cationic agent, mitochondrial targeting amino acid sequence) and enables enhanced electrophoretic transmembrane migration and up to 500‐fold accumulation of such agents in mitochondria.^9^, ^10^ When mitochondria become carcinogenic, they become dysfunctional: the ROS level and the mitochondrial membrane potential both increase. The higher mitochondrial transmembrane potential induces preferential accumulation and retention of cationic mitochondria targeting agents in cancer cells compared to normal cells.^7^, ^11^ Moreover, when mitochondria are photodamaged, they immediately lose their mitochondrial membrane potential and initiate apoptosis.^12^ Therefore, combining the PDT agent with the cationic mitochondrial targeting agent can result in rapid damage to cancer cells, improving therapeutic efficacy and reducing unwanted side effects.^13^

To establish an effective method for applying mitochondria‐targeting PS, several strategies have been researched, including adopting porphyrin‐based or nonporphyrin‐based PS, quantum dots, and metal‐based agents.^14^ However, these accompany problems: potential heavy metal properties, reduced therapeutic efficacy due to autofluorescence of the visible‐region dye, and a blood clearance problem, making biomedical applications more difficult.^15^ Near‐infrared (NIR) region PDT agents, containing cyanine dye and boron‐dipyrromethene (BODIPY) derivatives, have been developed to overcome these problems.^15^, ^16^ In NIR region PDT agents, the agents can be used to treat deep‐tissue level cancer due to the permeability of the NIR laser, and light scattering is reduced, thereby obtaining higher therapeutic efficacy.^17^ However, there is a disadvantage that singlet oxygen generation can be low when irradiated with an NIR laser.^17^ To address this issue, various methods to impart functionality to the dye have been studied, including heavy atom substitution into the dye structure to minimize the energy loss in excited states and increase the yield of singlet oxygen.^18^ Accordingly, by developing a novel PS that combines a functionalized NIR dye and a mitochondria‐targeting agent, it is possible to gain the benefit of rapid organelle clearance after treatment and also to remain in cancer mitochondria for a long time, amplifying the amount of ROS to the target sites irradiated by the laser. In addition, direct damage to mitochondria can induce therapeutic effects by apoptosis.

**Scheme 1:**
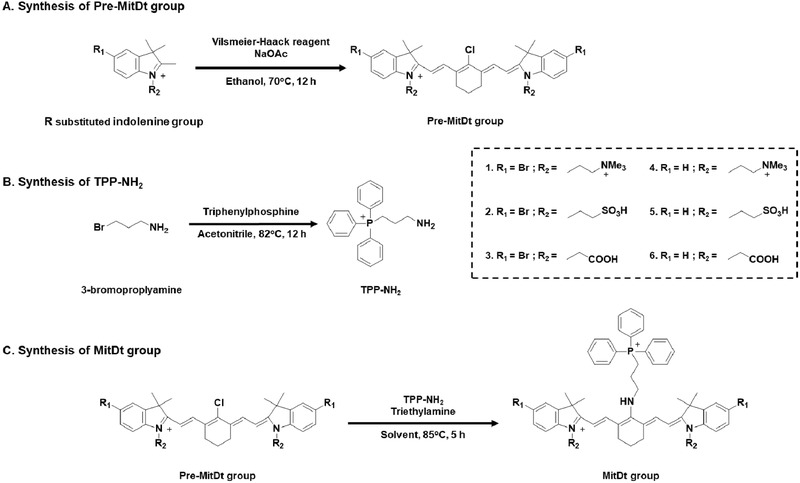
Structure and synthetic routes for MitDt groups. A) Precursor MitDt group (Pre‐MitDt group) was synthesized by substitution of R_1_ and R_2_ indolenine groups reacted with Vilsmeier–Haack reagent. B) TPP‐NH_2_ (3‐aminopropyl)triphenylphosphonium was synthesized by substitution of bromine in 3‐bromopropylamine with triphenylphosphine. C) The MitDt group was synthesized through the S_RN_1 mechanism of the pre‐MitDt group and the TPP‐NH_2_ group.

On this basis, we designed mitochondria‐targeting, brominated near‐infrared fluorophores for enhanced photodynamic cancer treatment. **Scheme** **1** illustrates the structure and synthetic routes of the mitochondria‐targeting photodynamic therapeutic agents (MitDt). The heptamethine meso‐position of the cyanine dye is bound to the 3‐aminomethyl(triphenylphosphonium) via the S_RN_1 mechanism, and the role of the fluorophore is to target the mitochondria. The R_2_ position affects the log*S* of the dye and is also designed to play a role in changing the net charge as the pH changes. The bromide, a halogen atom conjugated to the indolenine group, was designed to increase the amount of ROS produced by the heavy atom effect during laser irradiation. The synthesized MitDt derivative is relatively well internalized by cancer cells due to the potential of the cancer mitochondrial membrane and well retained for a long time, in contrast to other tissues. The uptake of the MitDt series into the cancer mitochondria released singlet oxygen when irradiated with a 662 nm laser, and the heavy atom effect of the bromine increased the amount of ROS produced, thereby enhancing the therapeutic effect on the cancer and reducing side effects. To demonstrate photoinduced apoptosis of MitDt compounds, in vitro and in vivo studies of MitDt compounds were carried out.

## 2 Results and Discussion

### 2.1 Synthesis and Characterization of Mitochondria‐Targeting Photodynamic Therapeutic Agents (Mitdt)

In order to establish mitochondrial targeting and PDT ability of PS, the cyanine dye compounds were composed of three parts. First, TPP was used to impart mitochondria targeting ability. Second, an R_2_ site was used to control solubility and charge in the dye structure. Finally, the heavy atom effect was used to enhance the photodynamic effect at R_1_ position. The detailed synthetic methods for the MitDt compounds are shown in Scheme 1. Briefly, chloro‐subsitituted cyanine dyes containing symmetric sulfonate, carboxylic acid, and quaternary ammonium cations substituents were prepared through salt concentration using Vilsmeier–Haack reagent. In order to conjugate TPP with meso‐chlorine atoms in the cyanine dye, the triphenylphosphonium moiety, substituted with an amine group, was then conjugated using the S_RN_1 displacement pathway.^19^ Synthesis of MitDt compounds was confirmed using ^1^H NMR and high‐resolution mass spectrometry (HR‐MS) methods.

After the synthesis, optical and chemical property experiments on the MitDt compounds were carried out after dissolving them in methanol at a concentration of 0.1 × 10^−6^ m. Regarding the absorbance and emission spectra relative to the pre‐MitDt group, the maximum absorbance was shifted from (≈780 ± 10 to 630 ± 10) nm after triphenylphosphonium was conjugated. The maximum emission wavelength was shifted by 70 ± 10 nm. Furthermore, a broad spectrum of absorption and emission was also confirmed. These results indicate that nitrogen atoms take part in the intermolecular charge transfer and engender competing resonance structures (Figure S1, Supporting Information).^19^

**Figure 1:**
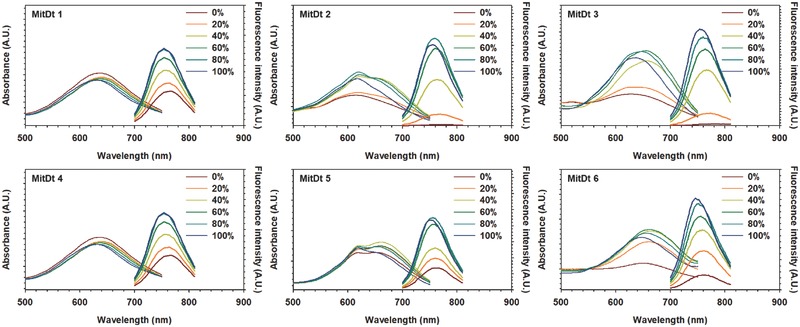
The dependence of methanol content (in water) on the absorption and emission spectra of MitDt compounds.

In addition, we investigated the absorption and emission spectra dependent on methanol concentration. The absorbance of quaternary ammonium substituents (MitDt‐1 and MitDt‐4) did not change according to the methanol ratio, but the fluorescence intensity decreased (**Figure** **1**). As shown in Figure S2 in the Supporting Information, the quaternary ammonium substituents (MitDt‐1 and MitDt‐4) show higher log*S* values and hydrophilicity than other substituents due to the permanent positive charge group, as shown in Table S1 in the Supporting Information. In addition, the decrease in fluorescence intensity of the quaternary ammonium substituents is due to aggregation caused quenching. The quaternary ammonium substituents have a tendency to aggregate due to the lipo‐cationic properties and pi–pi interaction of the TPP group.^20^ On the contrary, the absorbance and fluorescence intensity of sulfonate (MitDt‐2 and MitDt‐5) and carboxylic acid substituents (MitDt‐3 and MitDt‐6) increased with increasing methanol fraction due to lower log*S* values and neutral charge (Figure S2 and Table S1, Supporting Information). Furthermore, other chemical properties (polarizability, polar surface area, etc.) were also calculated using MarvinSpace (ChemAxon, Hungary) (Table S1, Supporting Information), providing a basis from which to investigate optimal compounds for photodynamic therapy.

### 2.2 ROS Generation Efficacy of Mitdt Compounds

**Figure 2:**
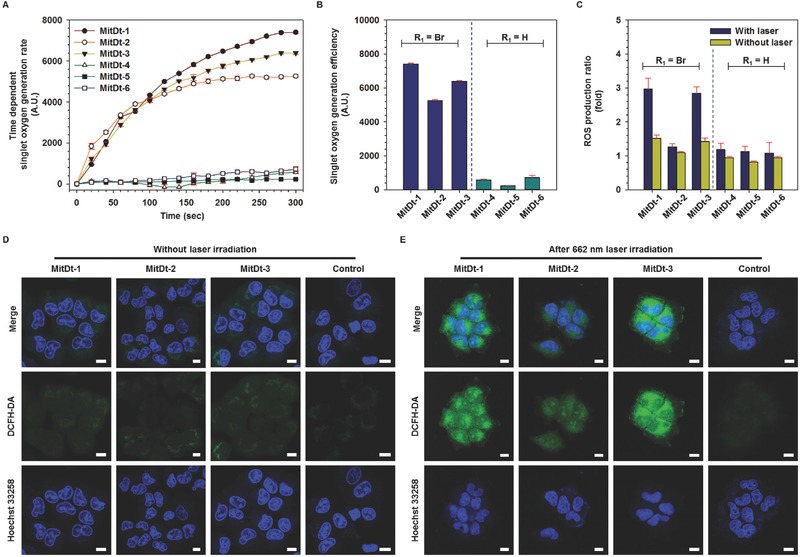
Photodynamic efficacy of MitDt compounds. Time‐dependent singlet oxygen generation rate for 5 min A) and singlet oxygen generation rate after 5 min irradiation B) of MitDt compounds (150 × 10^−6^ m) were detected using singlet oxygen sensor green upon NIR laser irradiation in DPBS. C) ROS production of MitDt compounds (10 × 10^−6^ m) in NCI‐H460 tumor cells compared to nontreated group using 2′,7′‐dichlorofluorescin diacetate (DCFH‐DA) without laser or after 5 min NIR laser irradiation. (D) and (E) are visualizations of in vitro ROS production level of brominated MitDt group using DCFH‐DA without laser, or after 5 min NIR laser irradiation in NCI‐H460 tumor cells measured by confocal laser scanning microscopy. A single irradiation from the NIR laser at 662 nm was given (100 mW cm^−2^ power density) in all the in vitro tests.

Before investigating photodynamic therapeutic efficacy, we compared the singlet oxygen production capacity and the ROS production capacity of the MitDt compounds. To confirm the singlet oxygen production of MitDt compounds, each compound was dissolved in Dulbecco's phosphate buffered saline (DPBS) containing 5% dimethyl sulfoxide (DMSO) to solubilize MitDt compounds at a concentration of 150 × 10^−6^ m; the amount of singlet oxygen produced by the laser irradiation (662 nm, 100 mW cm^−2^) was then checked using a singlet oxygen sensor green (SOSG) kit. Consequently, it was confirmed that the singlet oxygen production increased with laser irradiation time in the case of the brominated MitDt group (MitDt‐1, MitDt‐2, and MitDt‐3), but singlet oxygen production was not increased in the nonsubstituted MitDt group (MitDt‐4, MitDt‐5, and MitDt‐6) despite increased laser irradiation time (**Figure** **2**A). When the side chains of each R_2_ group are symmetrical, the singlet oxygen production of the brominated group (compared with the nonsubstituted groups) is as follows: quaternary ammonium: 12.83‐fold, sulfonate: 22.74‐fold, and carboxylic acid: 8.93‐fold. The singlet oxygen production is presented in the form of a sigmoid graph and shows a saturation value 5 min after laser irradiation (MitDt‐1: 7405.67, MitDt‐2: 5269.56, MitDt‐3: 6391.88, MitDt‐4: 577.02, MitDt‐5: 231.61, and MitDt‐6: 715.07) (Figure 2B).

We also measured fluorescence lifetime. Comparing the fluorescence lifetime of the brominated group with that of the protonated group, it was confirmed that the brominated group had short fluorescence lifetime (Pre‐MitDt‐1:0.29 ns, Pre‐MitDt‐4: 0.52 ns, Pre‐MitDt‐2: 0.49 ns, Pre‐MitDt‐4: 0.69 ns, Pre‐MitDt‐3: 0.45 ns, and Pre‐MitDt‐6: 0.57 ns), as shown in Figure S3 in the Supporting Information. The heavy atom nuclei cause spin–orbit coupling and enhanced intersystem crossing into the triplet state. Therefore, the heavy atoms have a short fluorescence lifetime because they excited the singlet state. Therefore, we can confirm that the difference in singlet oxygen production from the above comes from the heavy atom effect and increasing PDT efficiency of cyanine dyes.^21^

To confirm the in vitro ROS production of each compound, we first checked the cell uptake ability of each compound against human lung cancer NCI‐H460 and human breast cancer MCF‐7 cell lines (Figure S4A,B, Supporting Information). As a result, it was found that all the materials could be internalized by the cells in a short time. In these figures, the uptake efficiency of the brominated group is higher than that of the protonated group. These results illustrate that hydrophobicity of the bromine atom increases the log*P* value of the material. In addition, quaternary ammonium substituted groups showed better uptake than other groups because their net charge was +4. Subsequently, we performed experiments to quantify the ROS produced in vitro for each compound (10 × 10^−6^ m in Dulbecco's modified Eagle's medium, DMEM) after uptake to NCI‐H460 lung cancer cells and laser irradiation for 5 min. The ROS production rate was then measured using 2′,7′‐dichlorofluorescin diacetate (DCFH‐DA). At the same time, tests were also performed in which the cells were incubated with MitDt compounds but not irradiated with the NIR laser. The trend of the ratio of singlet oxygen generation was similar to that after laser irradiation. However, for MitDt‐2 ROS generation was similar to that of the nonsubstitution group after laser irradiation (Figure 2C). We then further investigated ROS production after uptake of brominated dye (MitDt‐1, MitDt‐2, and MitDt‐3) by cells in the MCF‐7 line (Figure S5, Supporting Information). The amount of ROS produced in the MCF‐7 cell line was similar to that of NCI‐H460. The reason for these results is that, in the case of MitDt‐1, the net charge was maintained at a constant value of +4 regardless of the pH, and the MitDt‐2 has a “0” net charge. On the other hand, MitDt‐3 had a value of “0” at pH 7.4 and 5.5, but a value of +2 at endosomal pH (3.5; see Table S1, Supporting Information). MitDt‐1 and MiDt‐3 had a positive net charge at pH 3.5 and 7.4 and could enter mitochondria as a result. MitDt‐2, on the other hand, had a neutral net charge in this pH range. As shown in Figure 2D and Figure S6 in the Supporting Information, when the laser was not irradiating, the DCFH‐DA signal of MitDt was weak, and this result was not different from that of the control group. However, when the laser was irradiating, the DCFH‐DA signal became stronger in MitDt‐1 and MitDt‐3 (Figure 2E and Figure S7, Supporting Information). This result is consistent with the data in Figure 2C and Figure S5 in the Supporting Information.

### 2.3 Intercellular and Mitochondrial Localization of MitDt Compounds

**Figure 3:**
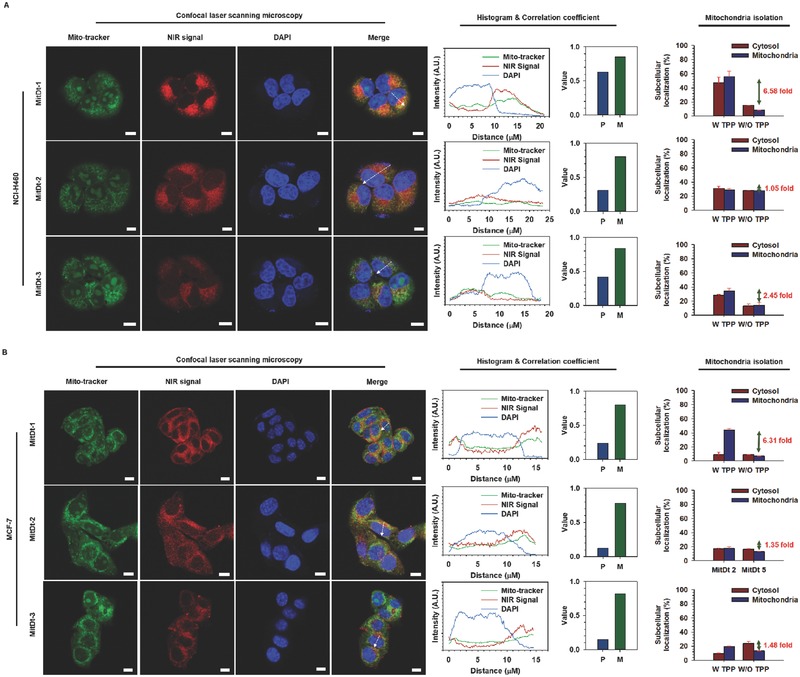
Subcellular localization of MitDt compounds. Mitochondrial localization of MitDt‐1, MitDt‐2, and MitDt‐3 was evaluated using confocal laser scanning microscopy. NCI‐H460 A) and MCF‐7 B) cancer cells were stained with mitotracker (MitoTracker Orange CMTMRos) and incubated. After that, both cell lines were incubated with MitDt‐1 and MitDt‐3, followed by fixation using 4% paraformaldehyde. Nuclei were counterstained with DAPI. The distances in the fluorescence profiles were indicated by dash lines (Scale bar = 10 µm). *P* (Pearson's correlation coefficient) and *M* (Manders overlap coefficient) between the Mito‐tracker and NIR signal were obtained using the ZEN2 program (Carl Zeiss, Germany). Quantification of MitDt compounds on mitochondria was confirmed using mitochondria isolation kit (W TPP: MitDt compounds, W/O TPP: Pre‐MitDt compounds).

In order to determine the subcellular localization of the selected MitDt compounds in the cancer cell, the position was detected using confocal laser scanning microscopy (CLSM) with 4′,6‐diamidino‐2‐phenylindole (DAPI), and MitoTracker after 3 h uptake of the MitDt compounds (**Figure** **3**). The lipophilic property of the TPP group site in both groups showed that localization in the mitochondria was successful. Among all compounds, MitDt‐1 was more colocalized with the mito‐tracker in two cancer cell lines than MitDt‐2 and MitDt‐3. In particular, we confirmed that MitDt‐1 entirely matched the mito‐tracker signal. On the other hand, MitDt‐2 and MitDt‐3 showed a tendency to match the mito‐tracker only slightly. Subsequently, to quantify the mitochondrial localization of the MitDt compounds, Mander's overlap coefficient (MOC) and Pearson's correlation coefficient (PCC) were calculated using the ZEN2 program. MOC has the following disadvantages: it does not reflect a substantial change and interpretation in data processing is difficult. However, it is possible to compare reciprocal association ratios for two fluorescent markers, although it is not as effective as PCC.^22^ All MOC values in Figure 3 were found to be between 0.5 and 1.0, which can be considered a positive range. Therefore, we confirmed the degree of efficiency of each correlation using a PCC method that only measured the correlation using independent signal levels and signal offsets. In the PCC method, there is a correlation for each signal when the value is 0.5 or less, and 0.5 or more.^23^ As shown in the PCC and MOC values, MitDt‐1 was more internalized by mitochondria than MitDt‐2 and MitDt‐3 in the other groups in both cell lines. This shows that a net charge of +4 was maintained regardless of the pH in the case of MitDt‐1; whereas in the case of MitDt‐2 and MitDt‐3, the pH had to be lowered to “5” or less, resulting in a slight positive charge. Therefore, when we checked the distribution of MitDt compounds in the cancer cell, MitDt‐1 is better accumulated due to its lipo‐cationic site and net charge than is MitDt‐2 and MitDt‐3. In addition, we confirmed the mitochondrial targeting ability of TPP group by using a mitochondria isolation assay (W TPP: MitDt compounds, W/O TPP: Pre‐MitDt compounds). Comparing the subcellular organelle uptake versus the washed cell uptake after 3 h treatment of each material, we found a cellular uptake similar to that shown in Figure S4 in the Supporting Information. In comparison with MitDt‐2 (NCI‐H460: 1.05‐fold, MCF‐7: 1.35‐fold) and MitDt‐3 (NCI‐H460: 2.45‐fold, MCF‐7: 1‐fold), MitDt‐1 has higher mitochondria uptake than Pre‐MitDt‐1 (NCI‐H460: 6.58‐fold, MCF‐7:6.31‐fold). The reason for this is that Pre‐MitDt‐1 has a highly positive charge and has water‐soluble properties, but it has low hydrophobicity and tends to adhere to the cell membrane. On the other hand, MitDt‐1 has a lipo‐cationic site (TPP position) and can be internalized to the mitochondria. MitDt‐2 and MitDt‐3 showed slightly increased mitochondria uptake compared to Pre‐MitDt but the water solubility was drastically lowered (precipitation occurred), thus lowering the bioavailability. In addition, the mitochondria localization efficiency of MitDt‐1 (NCI‐H460: 55.62%, MCF‐7: 43.81%) was much higher than that of other compounds; MitDt‐2 (NCI‐H460: 29.04%, MCF‐7: 17.47%), MitDt‐3 (NCI‐H460: 34.41%, MCF‐7: 19.26%). Based on the results, MitDt‐1 imbued remarkable mitochondria‐targeting ability. All things considered, our group selected MitDt‐1, composed of quaternary ammonium cations and brominated indolenine groups, as the therapeutic agent.

We next investigated the cellular uptake mechanism of MitDt‐1 by the NCI‐H460 and MCF‐7 cell lines using flow cytometry (Figure S8A,B, Supporting Information). Among the various cellular uptake mechanisms, the endocytotic mechanism was investigated after pretreatments with chloropromazine (CPZ, clathrin inhibitor), 5‐(*N*‐ethyl‐*N*‐isopropyl)‐amiloride (EIPA, macropinocytosis inhibitor) and methyl‐β‐cyclodextrin (MβCD, caveolae inhibitor), respectively. In both the NCI‐H460 and MCF‐7 cells, no significant difference was detected between treated and nontreated groups, indicating that MitDt‐1 was not internalized by cancer cells using the endocytic mechanism. We conducted an experiment after the former process, using 2‐deoxy‐d‐glucose (2‐DG, glycolysis inhibitor), sulfobromophthalein (BSP, organic‐anion transporting inhibitor, OATP), and hypertonic sucrose (HS, interference in the dispersion of clathrin lattices on the cellular membrane) to investigate the mechanism of uptake of MitDt‐1. These results show that the cellular uptake of MitDt‐1 was not associated with the glycolytic metabolic pathway or with the clathrin lattices on the cellular membrane. In addition, OATP is involved in transporting large and fairly hydrophobic organic anions. It was found that OATP inhibitor had no effect on the cancer cell uptake of MitDt‐1.^24^ However, we found that cell counts of MitDt‐1 were dramatically decreased after ice incubation in both cancer cell lines. These results demonstrated that MitDt‐1 does not bind nonspecifically to cancer cell surfaces due to positive charge but is most dominant in energy‐dependent cellular internalization and transmission into cancer mitochondria.^25^

### 2.4 In Vitro Photodynamic Therapeutic Efficacy of Mitdt‐1

**Figure 4:**
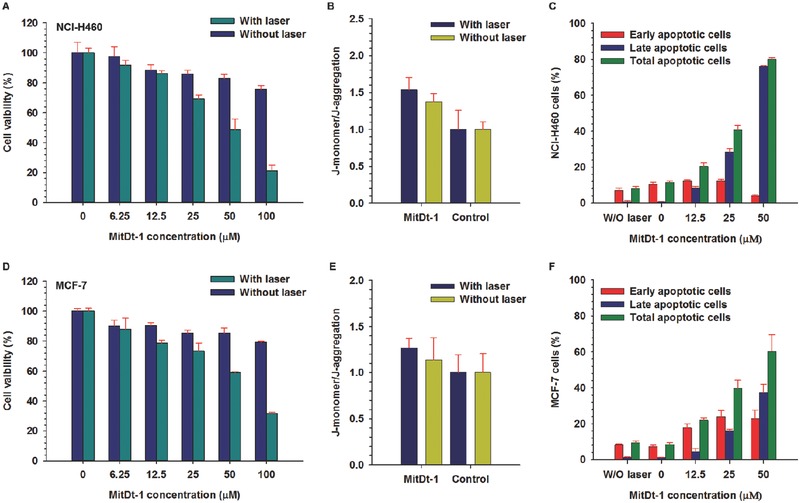
In vitro photoinduced therapeutic efficacy of MitDt‐1. Cell viability for NCI‐H460 A) and MCF‐7 D) were investigated using MTT assay. The relative mitochondrial membrane potential of NCI‐H460 B) and MCF‐7 E) cell lines were investigated using MitDt‐1 (35 × 10^−6^ m) and JC‐1 dye without or after NIR laser irradiation. The photodynamic therapeutic mechanisms in NCI‐H460 C) and MCF‐7 F) at different concentrations of MitDt‐1 were determined using flow cytometry. Cells were costained with propidium iodide (PI) and Annexin V‐FITC for flow cytometry analysis. A single 662 nm NIR laser irradiation was given (100 mW cm^−2^ power density) for 5 min in all the in vitro tests.

To confirm the photodynamic therapeutic efficacy of MitDt‐1 in vitro, we performed a cell viability test of MitDt‐1 using an 3‐(4,5‐dimethylthiazol‐2‐yl)‐2,5‐diphenyltetrazolium bromidefor (MTT) assay (**Figure** **4**A,D). It was confirmed that the MitDt‐1 concentration of (0–100) × 10^−6^ m in both the NCI‐H460 and MCF‐7 cell lines did not cause cytotoxicity. On the other hand, when the laser (662 nm, 100 mW cm^−2^) irradiated MitDt‐1 for 5 min under a corresponding concentration, the cell viability of (48.33 ± 7.39)% at 50 × 10^−6^ m and (21.18 ± 3.85)% at 100 × 10^−6^ m was observed in the NCI‐H460 cell line. The results for the MCF‐7 cell line were (58.95 ± 0.62)% at 50 × 10^−6^ m and (31.61 ± 0.85)% at 100 × 10^−6^ m. In addition, JC‐1 and an apoptosis assay can be used to confirm PDT induced apoptosis. The JC‐1 assay was performed using MitDt‐1 at a concentration of 35 × 10^−6^ m to confirm that the mechanism of the therapeutic effect was associated with mitochondria (Figure 4B,E). When mitochondria are healthy and not in a destabilized state, JC‐1 dye is localized in the mitochondria and becomes a JC‐1 aggregate form, whereas mitochondria that are unhealthy and destabilized produce a JC‐1 monomer form. Based on the nontreated group, both the laser‐treated MitDt‐1 group and the MitDt‐1 untreated group had the J‐monomer type. The reason for this is that in the case of the nonlaser‐irradiated group, the J‐monomer form appears because the TPP group and the charge on the material lower the potential of the mitochondrial membrane. In the case of the laser irradiated group in both cell lines, MitDt‐1 internalized into the mitochondria produces singlet oxygen and damages the mitochondria, inducing an apoptosis pathway of cell. In the case of the NCI‐H460, the mitochondria were better localized than MCF‐7, as shown in Figure 3, resulting in a more dramatic result.

Total apoptosis values (early apoptosis + late apoptosis) were determined using propidium iodide (PI) and Annexin V‐FITC to determine whether this therapeutic effect was from induction of apoptosis or necrosis by laser energy (Figure 4C,F).^26^ The apoptosis assay was performed by flow cytometry after laser irradiation (662 nm, 100 mW cm^−2^) according to the MitDt‐1 concentration. Total apoptosis values were (8.04 ± 1.21)% and (9.40 ± 1.05)%, respectively, when treated with the laser alone without MitDt‐1 treatment. As the MitDt‐1 concentration increased, the percentage of total apoptosis gradually increased, and it was found that the early apoptosis state slightly shifted to the late apoptosis state. Taken together, the results for the in vitro therapeutic effect of MitDt‐1 suggest that phototherapeutic efficacy is associated with mitochondria, and that cancer cell death occurs due to apoptosis.

### 2.5 In Vivo Photodynamic Therapeutic Efficacy of MitDt‐1

**Figure 5:**
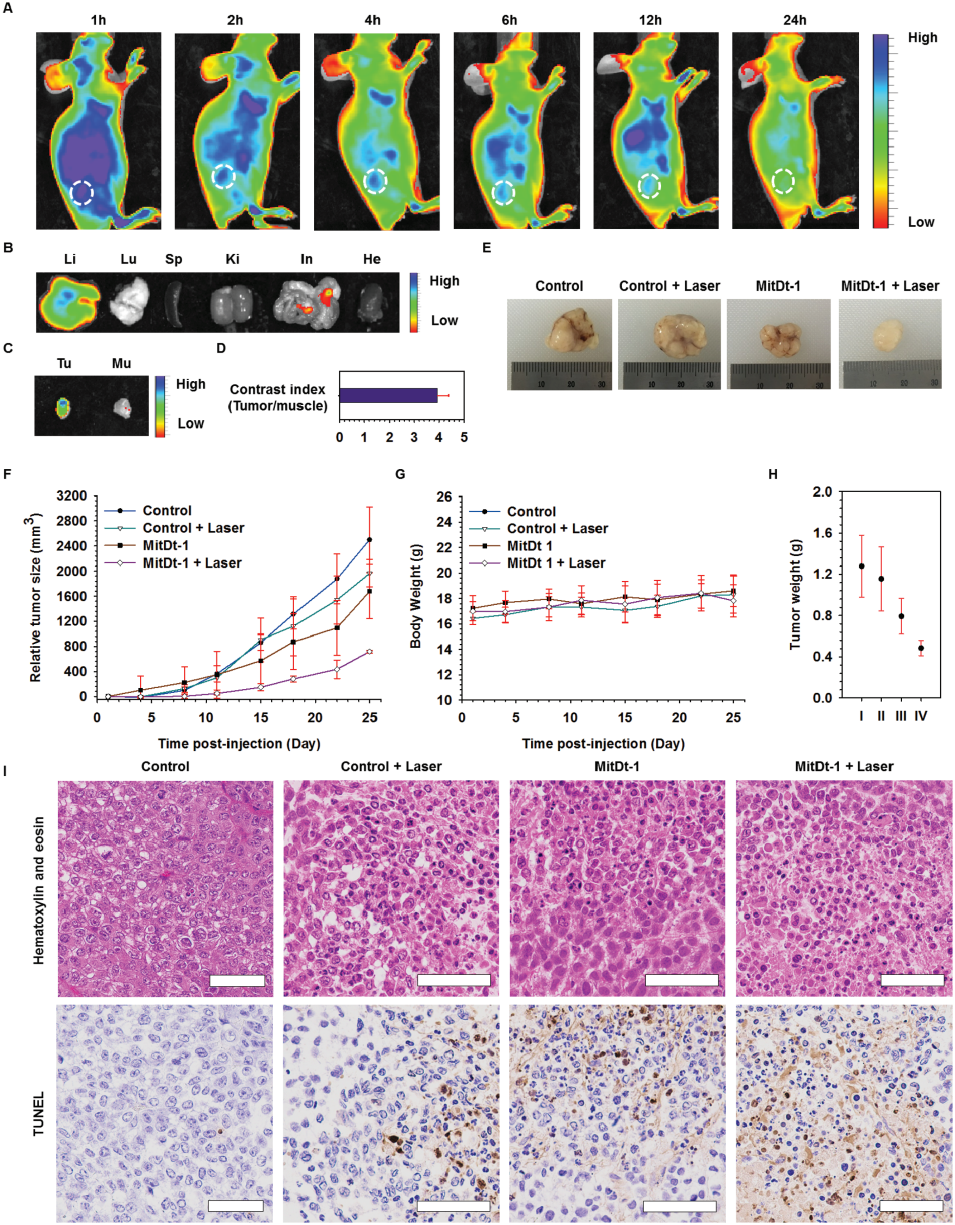
In vivo photoinduced therapeutic efficacy of MitDt‐1. NIR fluorescence images of NCI‐H460 tumor bearing mice over time A), and ex vivo NIR fluorescence images B, C) after sacrifice of mice 6 h after injection were measured using IVIS. The contrast index D) was obtained by dividing the tumor NIR signal by the muscle signal obtained from Figure 5C. To investigate the antiproliferative activity of MitDt‐1 with laser irradiation, four groups were created: control, control + laser, MitDt‐1, and MitDt‐1 + laser. 100 × 10^−6^ m of MitDt‐1 (200 µL) was injected in a tail vein into NCI‐H460 tumor bearing mice. One hour after injection, the laser was used to irradiate the tumor in the “+ laser” groups for 5 min. A) Change in relative tumor size (mm^3^) and B) body weights of HuCCT1 xenograft‐bearing mice were monitored at predetermined times for 26 d (*n* = 5, error bars represent the standard deviation). Tumor tissue sections were excised from different treatment groups of mice after the 26th day. H) Excised tumor weights from different treatment groups and E) excised NCI‐H460 tumor images. I) Histology analysis after hematoxylin and eosin staining and TUNEL assay was performed for the different treatment groups using a virtual microscope (Scale bar = 60 µm).

To confirm the in vivo applicability of MitDt‐1, NCI‐H460 cells were implanted into nude BALB/c female mice to form xenograft models. All animal experiments were carried out with the approval of the Animal Care Committee of the Korea Advanced Institute of Science and Technology (KAIST). In order to confirm the biodistribution of MitDt‐1 in mice, 200 µL of 100 × 10^−6^ m MitDt‐1 was injected into the tail vein when the tumor size reached 50 mm^3^. After the injection of MitDt‐1, the localization of MitDt‐1 over time showed that it was distributed throughout the body 1 h after injection. As the time elapsed after injection, rapid clearance of MitDt‐1 occurred in most organs; however, it was confirmed that the clearance of MitDt‐1 from the tumor occurred relatively slowly (**Figure** **5**A–C). Tumor mitochondria have a higher membrane potential compared to that in normal tissue. Therefore, in the TPP conjugation group, uptake by cancer during blood circulation leads to electrophoretic transmembrane migration and retention.^10^ In addition, we used the contrast ratio (tumor to muscle) to confirm the clearance of MitDt‐1. If the ratio exceeds 2.5, it means that relatively more MitDt‐1 remains in the tumor (Figure 5D).^15^, ^27^ As a result, it was found that the concentration ratio (tumor to adjacent muscle) was 3.94 ± 0.44; and thus it was confirmed that the MitDt‐1 was localized within tumor sites due to TPP. We checked the in vivo applicability of MitDt‐1 based on blood compatibility and H&E assays. First, to assess the adverse effect of MitDt‐1 on blood cells, a hemolytic activity test was performed with blood agar plates.^28^ We prepared 100 × 10^−6^ m of the MitDt‐1 (200 µL) to be used for in vivo experiments, treated with triton‐100, DPBS, and 200 µL of 100 × 10^−6^ m MitDt‐1 on a blood agar plate and incubated at 37 °C for 24 h. As shown in Figure S21 in the Supporting Information, an adverse effect of the MitDt‐1 treated group on blood cells compared to triton‐X100 (negative control) was not observed, meaning that MitDt‐1 is endowed with superior blood compatibility. We also performed H&E assays to evaluate the toxicity resulting fromMitDt‐1 (Figure S22, Supporting Information) after 24 h. And no severe damage in all the organs (including the heart, kidneys, spleen, lungs, and liver) was shown because an inappreciable difference between the DPBS and MitDt groups was exhibited in the H&E assay. These results indicated that MitDt‐1 was highly biocompatible even in in vivo systems and thus suitable for photodynamic therapeutic agents for in vivo applications. Thereafter, to demonstrate the antiproliferation of MitDt‐1 with laser irradiation, four test groups were prepared: Control, Control + laser, MitDt‐1, and MitDt‐1 + laser. 100 × 10^−6^ m of MitDt‐1 (200 µL) was injected in a tail vein. One hour after injection, the laser was used to irradiate the tumor in the + laser group for 5 min. After laser irradiation, the tumor size of each group and the body weight of the mice were measured every 3–4 d for four weeks (Figure 5F). First, we determined that there was no change in the body weight of the mice for four weeks, confirming that MitDt‐1 had no cytotoxicity (Figure 5G). When we compared changes in tumor size, there was growth in the tumors in the control group (up to 2499.90 ± 519.77 mm^3^). Similarly, the control + laser group and the MitDt‐1 group also showed growth in the tumor volume (1964.46 ± 218.23 and 1460.92 ± 221.52 mm^3^), respectively. On the other hand, in the group treated with laser after injection of MitDt‐1, the final volume was (719.41 ± 21.20) mm^3^. These results show that the group injected with MitDt‐1 inhibited tumor growth compared to the nontreated group. The biodistribution (Figure 5A) shows that MitDt‐1 stays in the tumors for a long time, whereas rapid clearance occurs from other parts of the body. Therefore, MitDt‐1, which is retained in cancer cells for a long time, destabilizes the mitochondria (Figure 4B), and provides a significant effect on the treated group by causing local damage due to ROS during laser irradiation. These results also confirmed that the tumor size and weight, obtained by sacrificing the mice at the end of the experiment, were significantly different (Figure 5E,H). In addition, to confirm the mechanism of tumor suppression in vivo, we treated one group with MitDt‐1 and did not treat another and sacrificed the mice 3 h later to obtain the tumors from each group. Paraffin sectioning was then performed to obtain a slide on one side, and H&E staining and transferase dUTP nick end labeling (TUNEL) assay were performed on the opposite sides (Figure 5I). Examination of the H&E stained slides for each treatment group showed that the tumors from the MitDt‐1 with the laser group were more damaged than were those in the control group. In addition, when the damage mechanism was analyzed by a TUNEL assay, it was confirmed that the tumor growth suppression was caused by apoptosis, as shown by the brown color indicating apoptosis. Taken together, these results indicate that MitDt‐1 is biocompatible and is applicable to in vivo systems, and that it is a platform for effective inhibition of tumor growth when irradiated with a laser.

## 3 Conclusion

In summary, MitDt‐1 was developed in this work as a PDT agent capable of targeting cancerous mitochondria. Synthesis of this photodynamic therapeutic agent was based on cyanine dye, and the agent was composed of a TPP moiety, quaternary ammonium, and brominated indolenine groups. The photochemical properties of the synthesized dye indicated (and were confirmed) to be sensitive to a wavelength in the NIR region, and the brominated dye group had a high singlet oxygen production rate. Moreover, MitDt‐1 accumulated in the mitochondria of the human cancer cell lines (NCI‐H460 and MCF‐7) via charge and lipocationic properties at TPP sites. We could also confirm that ROS production was amplified according to the on–off state of the 662 nm single laser, proving that intensive and effective photodynamic therapy is possible for the target cancer in vitro and in vivo by mitochondria destabilization apoptosis. Consequently, MitDt‐1 allowed outstanding phototherapy and is a promising structure for a cyanine dye based PDT system for cancer therapy.

## 4 Experimental Section

*Materials: N*,*N*‐dimethylformamide anhydrous, 1,3‐propane sultone, 3‐bromopropionic acid, 2,3,3‐trimethylindolenine, (3‐bromopropyl)trimethylammonium bromide, phosphorus pentachloride, acetic acid, 4‐bromophenylhydrazine hydrochloride, DPBS (10 × 10^−3^ m, pH 7.4), and DMEM were obtained from Sigma‐Aldrich. Triphenylphosphine, toluene anhydrous, methyl tert‐butyl ether, 3‐bromopropylamine hydrobromide, acetonitrile anhydrious, and triethylamine were obtained from alfa‐aesar. Aniline and cyclohexanone were obtained from Tokyo Chemical Industry. Ethanol, methanol, diethyl ether, sodium acetate, tetrahydrofuran, *n*‐hexane, and ethyl acetate were obtained Daejung chemical and metal. All chemicals were used without further purification. All reactions were performed under argon and monitored by thin layer chromatography.

*Chemical and Optical Properties*: Molecular weight, net charge at pH 7.4, Log*D* at pH 7.4, Log*P*, polarizability, polar surface area, hydrogen bonding donors/acceptors, and Hu˝ckel molecular orbital π energy were calculated using Marvin and JChem calculator plug‐ins (ChemAxon). The all of absorbance and emission spectrum of the MitDt dye group dissolved in methanol were measured using multimode microplate readers (Infinite 200 PRO, Tecan Group, Ltd., Switzerland). The fluorescence lifetime of MitDt compounds were confirmed using Fluorescence Lifetime Spectrometer (FL920, Edinburgh Instruments, UK) equipped with 785 nm laser.

*Singlet Oxygen Generation Efficiency*: The singlet oxygen generation efficiency of MitDt compounds was determined using SOSG (Thermo Fisher Scientific Inc., USA) kit. MitDt dye group (150 × 10^−6^ m) and SOSG (10 × 10^−6^ m) were mixed in DPBS (5% DMSO) and exposed to an NIR laser (662 nm, 100 mW cm^−2^) in time‐dependent manner. The fluorescence intensity of the mixture was immediately detected using spectrophotometer (excitation/emission: 504/525 nm). The singlet oxygen generation ratio was calculated as “*x*‐fold” in relation to the fluorescence intensity at “0” min irradiation.

*Cellular Uptake Efficiency*: Cells (2 × 10^4^ cells well^−1^) of the NCI‐H460 and MCF‐7 lines were seeded and cultured overnight at 37 °C in 5% CO_2_. The cells were treated with the same concentration of the MitDt dye group (10 × 10^−6^ m) for 3 h at 37 °C in 5% CO_2_, and the cells were washed with DPBS. Subsequently, the cells were incubated with radio immuno precipitation assay (RIPA) buffer (200 µL) for 10 min at 4 °C. The fluorescence of each lysate was detected with a spectrophotometer (excitation/emission: 640/760 nm). Cellular uptake efficiency was calculated based on the initial fluorescence.

*Intracellular ROS Level in Cancer Cells*: Intracellular ROS levels in cancer cells were detected using DCFH‐DA. Cells (2 × 10^4^ cells well^−1^) of the NCI‐H460 and MCF‐7 lines were seeded and incubated overnight at 37 °C in 5% CO_2_. Then, the cells were treated with same concentrations of MitDt dye group (10 × 10^−6^ m) for 3 h, and the cells were washed with DPBS. Subsequently, DCFH‐DA solution (10 × 10^−6^ m) was added to the cell for 1 h at 37 °C in 5% CO_2_ and then irradiated by an NIR laser (662 nm, 100 mW cm^−2^) for 5 min. At the same time, another group of cells was incubated and treated with MitDt but without NIR laser irradiation. For quantification of the ROS level, the cells were washed using DPBS, and fluorescence intensity was measured using spectrophotometer (excitation/emission: 495/515 nm) after adding RIPA buffer. The nontreated groups, with or without NIR irradiation, served as comparison. For visualization of the brominated MitDt dye group, the cell nuclei were stained using Hoechst 33258 for 10 min at 37 °C. Finally, the intracellular ROS level was monitored by confocal microscopy (ZEISS LSM 800, Carl Zeiss AG, Germany).

*Subcellular Localization*: Subcellular localization of MitDt‐1, MitDt‐2, and MitDt‐3 were determined by costaining with Mito‐tracker (500 × 10^−9^ m, MitoTracker Orange CMTMRos, Thermo Fisher Scientific, Inc., USA) and 4′,6‐diamidino‐2‐phenylindole, dilactate (DAPI, Thermo Fisher Scientific, Inc., USA). Cells (3 × 10^4^ cells well^−1^) of the NCI‐H460 and MCF‐7 lines were seeded on cell culture slides (SPL Life Sciences Co., Ltd., Korea) and incubated overnight at 37 °C in 5% CO_2_. Both cell lines were stained with Mito‐tracker incubated for 30 min at 37 °C in 5% CO_2_. Thereafter, each cell line was washed with DPBS; then, MitDt‐1 and 3 dissolved in DMEM (10 × 10^−6^ m) were added to each, respectively. After 3 h, the treated cells were fixed using 4% paraformaldehyde (PFA) at 37 °C for 15 min, and nuclei were counterstained with DAPI for 10 min at 37 °C. Subsequently, each medium was replaced with fresh DPBS, and subcellular localizations of MitDt dyes were monitored using confocal microscopy. Pearson's correlation coefficient and Mander's correlation coefficient were calculated using the ZEN2 program. The quantification of MitDt at mitochondria was confirmed by using mitochondria isolation kit (Thermo Fisher Scientific, USA) followed manufacturing processes.

*Cellular Uptake Mechanism*: Cells (5 × 10^4^ cells well^−1^) of the NCI‐H460 and MCF‐7 lines were seeded and cultured overnight at 37 °C in 5% CO_2_. After that, the cells were treated with various agents dissolved in DMEM as follows: CPZ (30 × 10^−6^ m for 1 h), methyl‐β‐cyclodextrin (MβCD, 10 × 10^−3^ m for 1 h), EIPA (20 × 10^−6^ m for 1 h), BSP (250 × 10^−3^ m for 5 min), 2‐DG (150 × 10^−3^ m for 45 min), and HS (0.4 m for 1 h). After pretreatments, the cells were washed with DPBS and further incubated with MitDt‐1 (10 × 10^−3^ m) in DMEM for 1 h at 37 °C in 5% CO_2_. To estimate the energy‐dependent process, the cells were incubated with MitDt‐1 for 1 h at 4 °C. After incubation, the cells were washed twice, and the cells collected were resuspended in 4% PFA (300 µL); then monitored using flow cytometry (FACSCalibur, Becton Dickinson and Co., USA).

*Photodynamic Therapeutic Efficacy*: The in vitro photodynamic therapeutic efficacy of MitDt‐1 was determined using the MTT assay. Cells (1 × 10^4^ cells well^−1^) of the NCI‐H460 and MCF‐7 lines were seeded and cultured overnight at 37 °C in 5% CO_2_. Then, the cells were treated with at different concentrations (0, 6.25 × 10^−6^, 12.5 × 10^−6^, 25 × 10^−6^, 50 × 10^−6^, and 100 × 10^−6^ m) of MitDt‐1 for 3 h at 37 °C in 5% CO_2_. After this time, the cells were irradiated with the NIR laser (662 nm, 100 mW cm^−2^) for 5 min. At the same time, cells were incubated and treated with the same MitDt‐1 concentration without NIR laser irradiation. Thereafter, the cells were further incubated for 24 h with fresh DMEM supplemented with 4% (v/v) fetal bovine serum (FBS). Then, the cells were washed with DPBS and treated with the MTT solution at 37 °C in 5% CO_2_. After 3 h, the resultant formazan crystals were solubilized in DMSO. The absorbance of the resulting solution was measured at 575 nm using multimode microplate readers.

*JC‐1 Assay*: Cells (5 × 10^4^ cells well^−1^) of the NCI‐H460 and MCF‐7 lines were seeded and cultured overnight at 37 °C in 5% CO_2_. Then the cells were treated with 35 × 10^−6^ m of MitDt‐1 for 3 h at 37 °C in 5% CO_2._ After this, the cells were irradiated with the NIR laser (662 nm, 100 mW cm^−2^) for 5 min. At the same time, the cells incubated and treated with the same concentration of MitDt‐1 without NIR laser irradiation was also performed. The cells were washed with DPBS, and fresh medium containing JC‐1 dye (100 µg mL^−1^) added, after which the culture was subjected to an additional 1 h of incubation. The cells were washed with DPBS, and the fluorescence of each group was detected using a spectrophotometer. JC‐1 monomer signals were obtained at 490/520 nm (excitation/emission), and JC‐1 aggregation signals were obtained at 550/610 nm (excitation/emission). Each J‐monomer/J‐aggregation value was expressed as the value obtained divided by the control value.

*Apoptosis Detection*: The different types of cell death were identified by staining with Annexin V and propidium iodide. Cells (3 × 10^4^ cells well^−1^) of the NCI‐H460 and MCF‐7 lines were seeded and cultured overnight at 37 °C in 5% CO_2_. Then, the cells were treated with different concentrations (0, 12.5 × 10^−6^, 25 × 10^−6^, and 50 × 10^−6^ m) of MitDt‐1 for 1 h at 37 °C in 5% CO_2._ After this, the cells were irradiated with the NIR laser (662 nm, 100 mW cm^−2^) for 5 min. At the same time, other cells were incubated and treated with the same concentration of MitDt‐1 without NIR laser irradiation. Then, the cells were further incubated for 3 h with fresh DMEM supplemented with 4% (v/v) of FBS before being collected. Finally, the cells were costained with CF488A Annexin V and propidium iodide (Annexin V and PI Apoptosis Kit, Biotium, Inc., USA) and analyzed using flow cytometry.

*In Vivo Therapeutic Efficacy*: All animal experiments were carried out with the approval of the Animal Care Committee of the KAIST. The xenograft model was prepared through the implantation of NCI‐H460 cells (1 × 10^7^ cells with 200 mL of DMEM) by subcutaneous injection into female BALB/c nude mice, which were 5–6 weeks old. When the tumor size of each mouse reached 30–50 mm, two groups of tumor‐bearing mice (*n* = 5) were injected through the tail vein with 100 × 10^−6^ m of MitDt‐1 (200 µL). At a predetermined time interval, the mice were anesthetized with isoflurane, and whole body images were provided using IVIS Lumina. At the end of the biodistribution experiment, the mice were sacrificed to collect their tumors and other organs. The in vivo and ex vivo NIR fluorescence levels were determined using Living Image (PerkinElmer, USA). To measure tumor inhibition efficacy, two mice groups (control + laser, MitDt‐1 + laser) were irradiated with the laser for 5 min (662 nm, 100 mW cm^−2^). The tumor sizes and body weights of the mice were measured at 3 or 4 d intervals. The length of the minor axis (2a) and the major axis (2b) of each tumor was measured using a caliper. The volume of each tumor was then calculated using the formula for a prolate spheroid [(4/3) × π × *a* ^2^ *b*]. The relative tumor volume was calculated by subtracting the initial tumor volume from the volume on the last day measured. Four hours after treatments, the tumors were paraffin‐sectioned and stained using hematoxylin and eosin (H&E), and then subjected to terminal deoxynucleotidyl TUNEL according to the manufacturer instructions. Stained tissue sections were analyzed using a virtual microscope (Aperio AT2, Leica Biosystems, Germany).

## Conflict of Interest

The authors declare no conflict of interest.

## Supporting information

Supplementary
