# Supplementary material for: Enhanced Photodynamic Cancer Treatment by Mitochondria‐Targeting and Brominated Near‐Infrared Fluorophores
Source: Adv Sci (Weinh). 2017 Dec 19;5(3):1700481. doi: 10.1002/advs.201700481 (PMC5867131; doi:10.1002/advs.201700481)
Supplement: Supplementary file 1 — Supplementary [file ADVS-5-1700481-s001.pdf]

Copyright WILEY-VCH Verlag GmbH & Co. KGaA, 69469 Weinheim, Germany, 2017.

ADVANCED SCIENCE
Open Access

Supporting Information

for *Adv. Sci.,* DOI: 10.1002/advs.201700481

Enhanced Photodynamic Cancer Treatment by Mitochondria-Targeting and Brominated Near-Infrared Fluorophores

*Ilkoo Noh, DaeYong Lee, Heegon Kim, Chan-Uk Jeong, Yunsoo Lee, Jung-Oh Ahn, Hoon Hyun, Ji-Ho Park, and Yeu-Chun Kim**

Copyright WILEY-VCH Verlag GmbH & Co. KGaA, 69469 Weinheim, Germany, 2017.

Supporting Information

Enhanced photodynamic cancer treatment by mitochondria-targeting and brominated near-infrared fluorophores

*Ilkoo Noh, DaeYong Lee, Heegon Kim, Chan-Uk Jeong, Yunsoo Lee, Jung-Oh Ahn, Hoon Hyun, Ji-Ho Park, and Yeu-Chun Kim **

Synthesis of the Pre-MitDt group: The 5-bromo-2,3,3-trimethyl-3H-indole and R substituted indolenine groups were prepared in accord with previous literature.[1] The prepared trimethylindolenine groups were reacted with 1,3-propane sultone and alkylating agent, (3-bromopropyl)trimethylammonium bromide and 3-bromopropionic acid in ACN for 48 h at 82 °C. After the mixture was cooled, the solvent was precipitated with toluene and washed with ether to afford a deep red product, which was used in the next step without further purification. The R substituted indolenine groups were reacted with Vilsmeier-Haack reagent (0.5 eq. with respect to the R substituted indolenine group) and sodium acetate in ethanol for 12 h at 70 °C.[2] The reaction mixture was cooled to room temperature; then precipitated in diethyl ether, and purified using preparative HPLC on a symmetry C18 column (5μm, 30 × 100 mm) at 25 °C. The mobile phase was water-ethanol in gradient mode. The MitDt group was used after confirming the peak detected at excitation wavelength 780 nm and emission wavelength 800 nm.

Synthesis of the MitDt group: The various synthesized pre-MitDt groups were reacted with (3-aminopropyl)triphenylphosphonium (TPP-NH_2) and TEA (3 eq. with respect to Pre-MitDt group) in DMSO (procedure 1) and DMSO/methanol (procedure 2) (1:1,v/v) 85 °C for 5 h.[3] The reaction mixture was precipitated in diethyl ether, and purified using preparative HPLC on a Symmetry C18 column (5 μm, 30 × 100 mm) at 25 °C. The mobile phase was water-methanol with 0.1% TFA (v/v) in gradient mode. The MitDt group was used after confirming the peak detected at excitation wavelength 640 nm and emission wavelength 750 nm. The purified MitDt fluorophores were analyzed using ultra-performance liquid chromatography (UPLC, Waters, USA) equipped with micrOTOF-Q II (Bruker, Germany) and structures were confirmed using ^{1}H NMR (Agilent 400MHz 54mm NMR DD2, Agilent, USA); see Figure S9-S20.

MitDt-1. Procedure 1 (brownish gold, yields: 30%). ^{1}H NMR (400 MHz, DMSO-d6, δ): 8.2 ppm (s, 2 H), 8.00-7.75 ppm (m, 21 H), 5.8 (d, J=12.7 Hz, 2 H), 3.99 (t, J = 7.0 Hz, 4 H),

3.81 – 3.70 (m, 6 H), 3.56 (m, 2 H), 3.01 (m, 2 H), 3.12 (s, 18 H), 2.89 (t, J=7.0, 2 H), 2.73 (m, 6H), 1.90 – 1.81 (m, 2 H), 1.54 (s, 12 H).

MitDt-2. Procedure 2 (Dark blue, yields: 21%). ^{1}H NMR (400 MHz, DMSO-d6, δ): 8.28 ppm (br-s, 2 H), 8.00-7.54 ppm (m, 21 H), 5.64 (d, J=12.7 Hz, 1 H), 5.53 (d, J=12.7 Hz, 1 H), 4.70 (t, J = 7.0 Hz, 4 H), 3.78 (m, 2 H), 3.59 (m, 2 H), 2.00 – 1.70(m, 2 H), 1.60 (m, 2 H), 1.47 (m, 12 H).

MitDt-3. Procedure 1 (Dark blue, yields: 14%). ^{1}H NMR (400 MHz, DMSO-d6, δ): 8.30 ppm (br-s, 2 H), 8.00-7.54 ppm (m, 21 H), 5.63 (d, J=13.3 Hz, 1 H), 5.11 (d, J=13.3 Hz, 1 H), 3.760 (t, J = 7.0 Hz, 4 H), 3.65 (m, 2 H), 3.08 (m, 2 H), 2.44 – 2.36(m, 4 H), 1.50 (m, 2H), 1.43 (m, 2 H), 1.33 (m, 12 H), 1.17 (m, 2 H), 1.47-1.40 (m, 2 H).

MitDt-4. Procedure 1 (Brownish gold, yields: 30%). ^{1}H NMR (400 MHz, DMSO-d6, δ): 8.16 ppm (s, 2 H), 8.00-7.75 ppm (m, 23 H), 5.8 (d, J=12.7 Hz, 2 H), 4.12 (t, J = 7.3 Hz, 4 H), 3.74 – 3.71 (m, 6 H), 3.0 (m, 2H), 3.4 (m, 2 H), 3.12 (s, 18 H), 3.01 (t, J=7.3, 2 H), 2.89 (m, 4 H), 2.73 (m, 2 H), 1.90 – 1.83 (m, 2 H), 1.51 (s, 12 H).

MitDt-5. Procedure 2 (Dark blue, yields: 17%). ^{1}H NMR (400 MHz, DMSO-d6, δ): 8.56 ppm (br-s, 2 H), 8.00-7.54 ppm (m, 23 H), 5.67 (d, J=13.4 Hz, 1 H), 5.57 (d, J=13.4 Hz, 1 H), 4.78 (t, J = 7.0 Hz, 4 H), 3.76 (m, 2 H), 3.66 (m, 2 H), 2.12 - 1.82 (m, 4H), 1.55 (m, 2 H), 1.45 (m, 12 H).

MitDt-6. Procedure 1 (Dark blue, yields: 15%). ^{1}H NMR (400 MHz, DMSO-d6, δ): 8.26 ppm (br-s, 2 H), 8.00-7.54 ppm (m, 23 H), 5.63 (d, J=12.5 Hz, 1 H), 5.11 (d, J=12.5 Hz, 1 H), 3.84 (t, J = 7.0 Hz, 4 H), 3.77 (m, 2 H), 3.3 (m, 2 H), 2.44 – 2.36(m, 4 H), 1.56 (m, 2H), 1.49 (m, 2 H), 1.47-1.40 (m, 2 H), 1.37 (m, 12 H), 1.21 (m, 2 H).

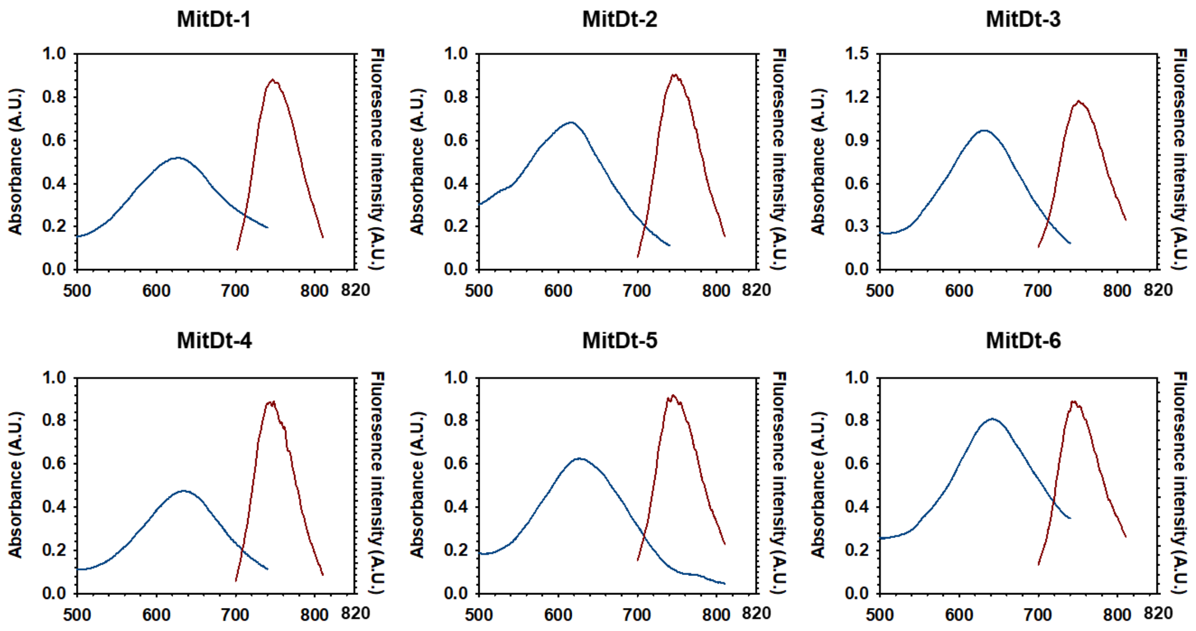

Property	MitDt-1	MitDt-2	MitDt-3	MitDt-4	MitDt-5	MitDt-6
λ_{max} (nm)	627	618	630	636	626	641
λ_{em} (nm)	746	748	750	754	746	752
Stoke shift (nm)	119	130	120	118	120	111

Figure S1. Excitation and emission spectra of MitDt compounds in methanol.

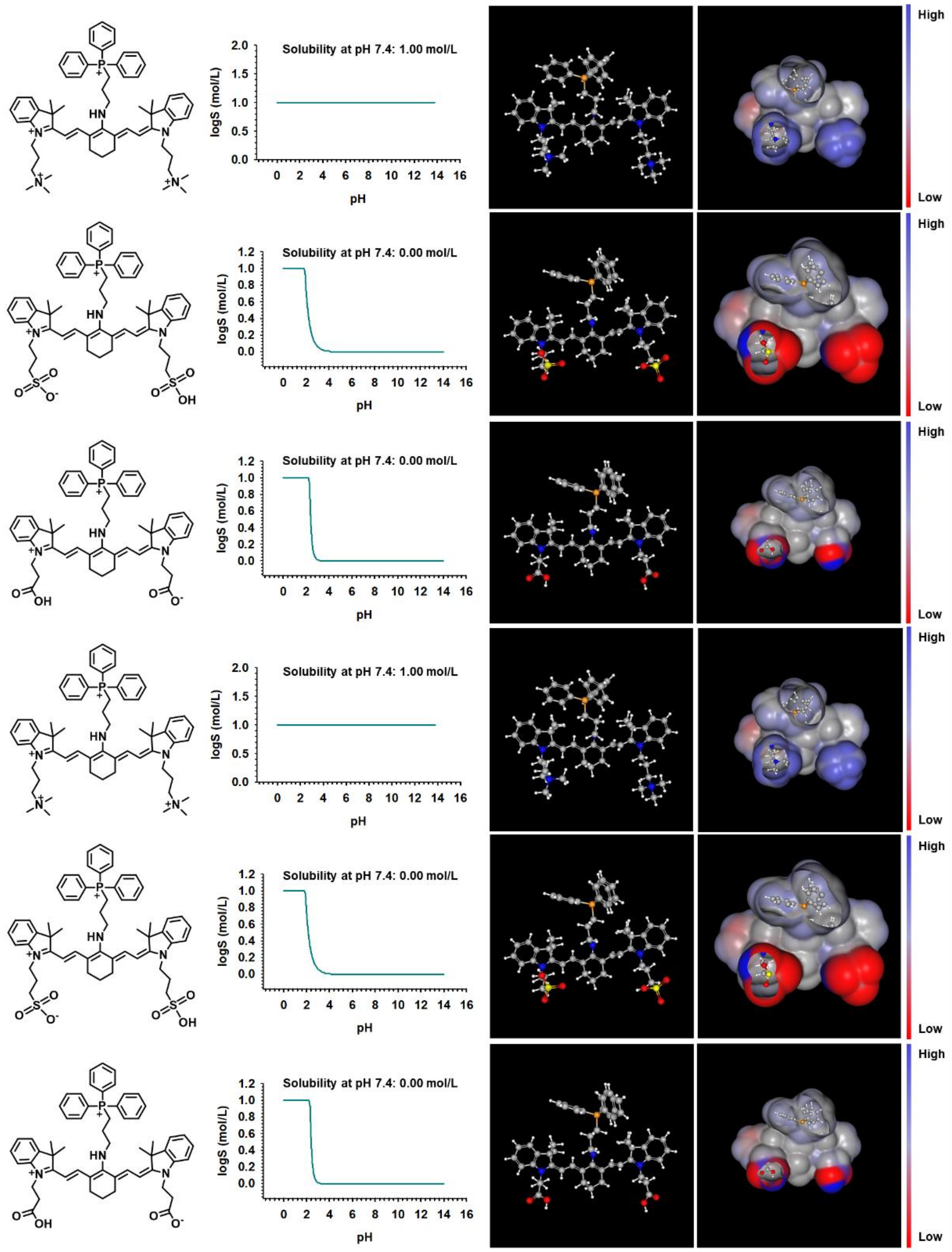

Figure S2. Hydrophobicity map and pH dependent solubility parameters of MitDt compounds: all the data were calculated using MarvinSpace. (ChemAxon)

Table S1. Comparison of chemical properties of various cyanine dye derivatives: All the parameters were calculated using the Marvin and JChem calculator plug-ins. (ChemAxon).

Properties	MitDt-1	MitDt-2	MitDt-3	MitDt-4	MitDt-5	MitDt-6
MW [Da]	1098.15	1142.05	1041.90	940.356	984.26	884.113
Net charge [pH 7.4]	+ 4	0	0	+ 4	0	0
Net charge [pH 5.5]	+ 4	0	0	+ 4	0	0
Net charge [pH 3.5]	+ 4	0	+ 2	+ 4	0	+ 2
Log P	5.52	11.55	13.12	3.99	10.31	11.58
Polarizability	122.74	117.64	109.09	115.05	110.04	101.59
Polar surface area [$Å^2$]	22.86	137.26	103.12	22.86	137.26	103.12
Hydrogen bonding donors / acceptors [pH 7.4]	1.95 / 7.05	1.95 / 21.05	1.95 / 17.05	1.95 / 1.05	1.95 / 15.05	1.95 / 11.05
Hückel molecular orbital π energy [pH 7.4]	59.38	77.19	71.50	59.38	77.19	71.50

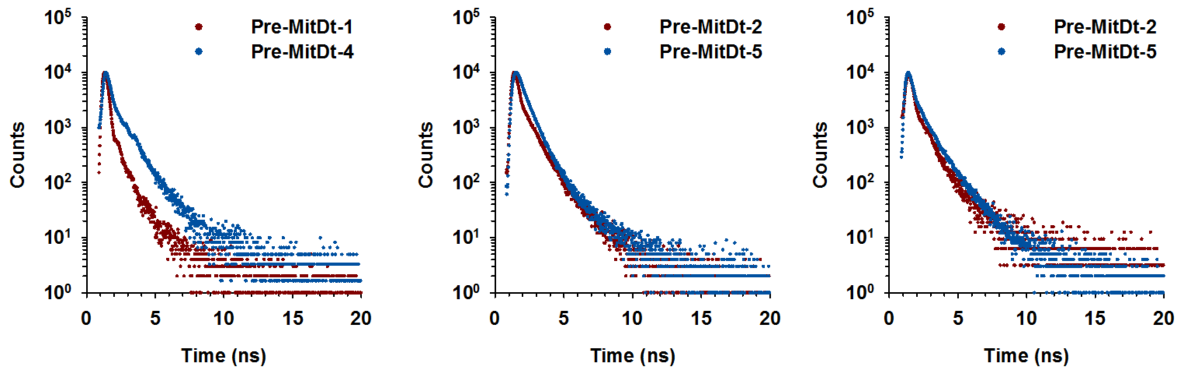

Figure S3. Fluorescence lifetime of Pre-MitDt compounds at solid state.

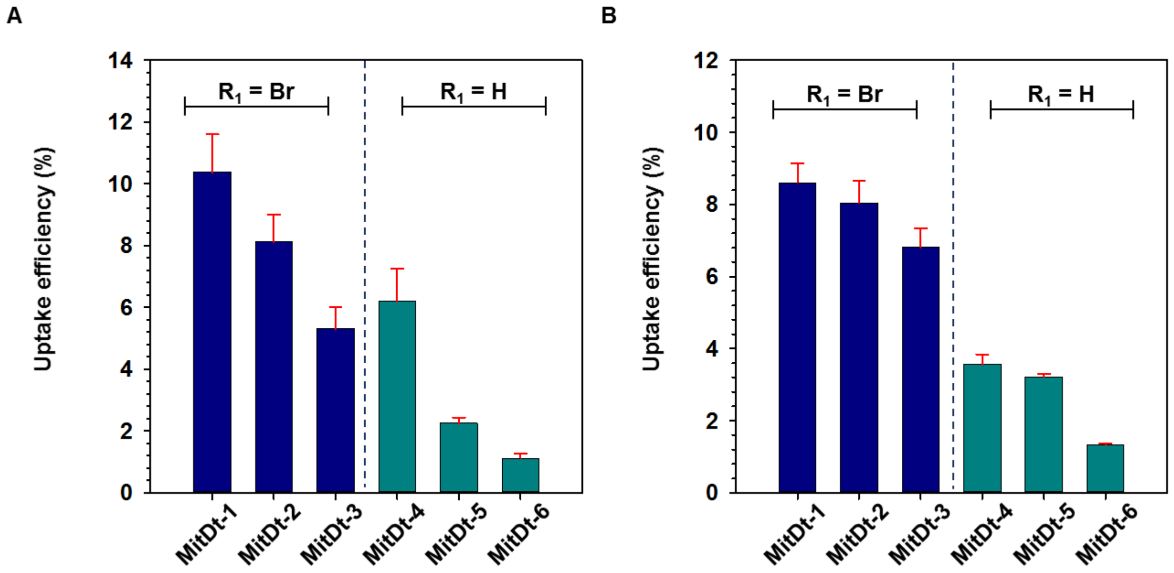

Figure S4. Cellular uptake efficiency of MitDt compounds (10 μM) in NCI-H460 (A), MCF-7 (B) cell lines after 3 h of uptake.

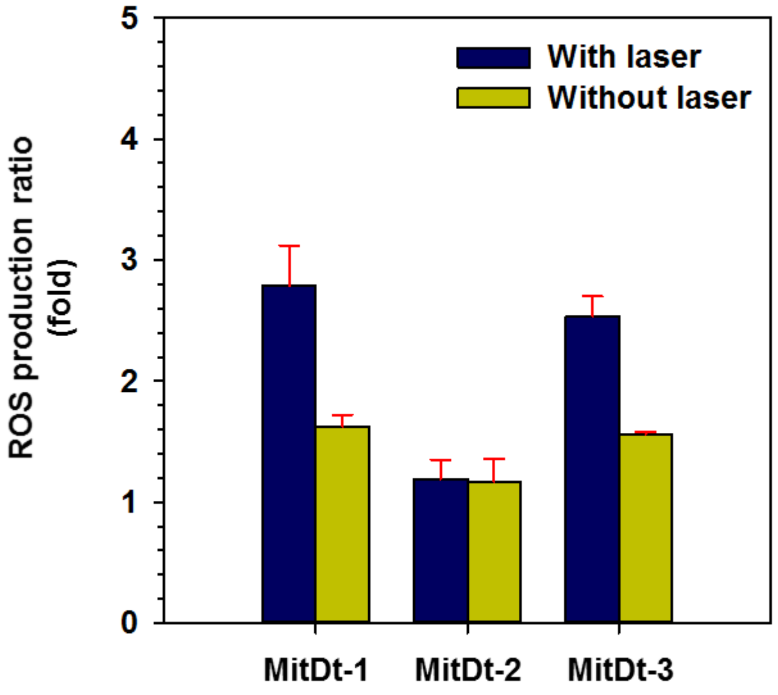

Figure S5. ROS production of brominated MitDt compounds (10 μM) in MCF-7 cells compared to a non-treated group using 2′,7′-Dichlorofluorescin diacetate (DCFH-DA) without laser, or after 5 min NIR laser irradiation. (100 mW/cm^2)

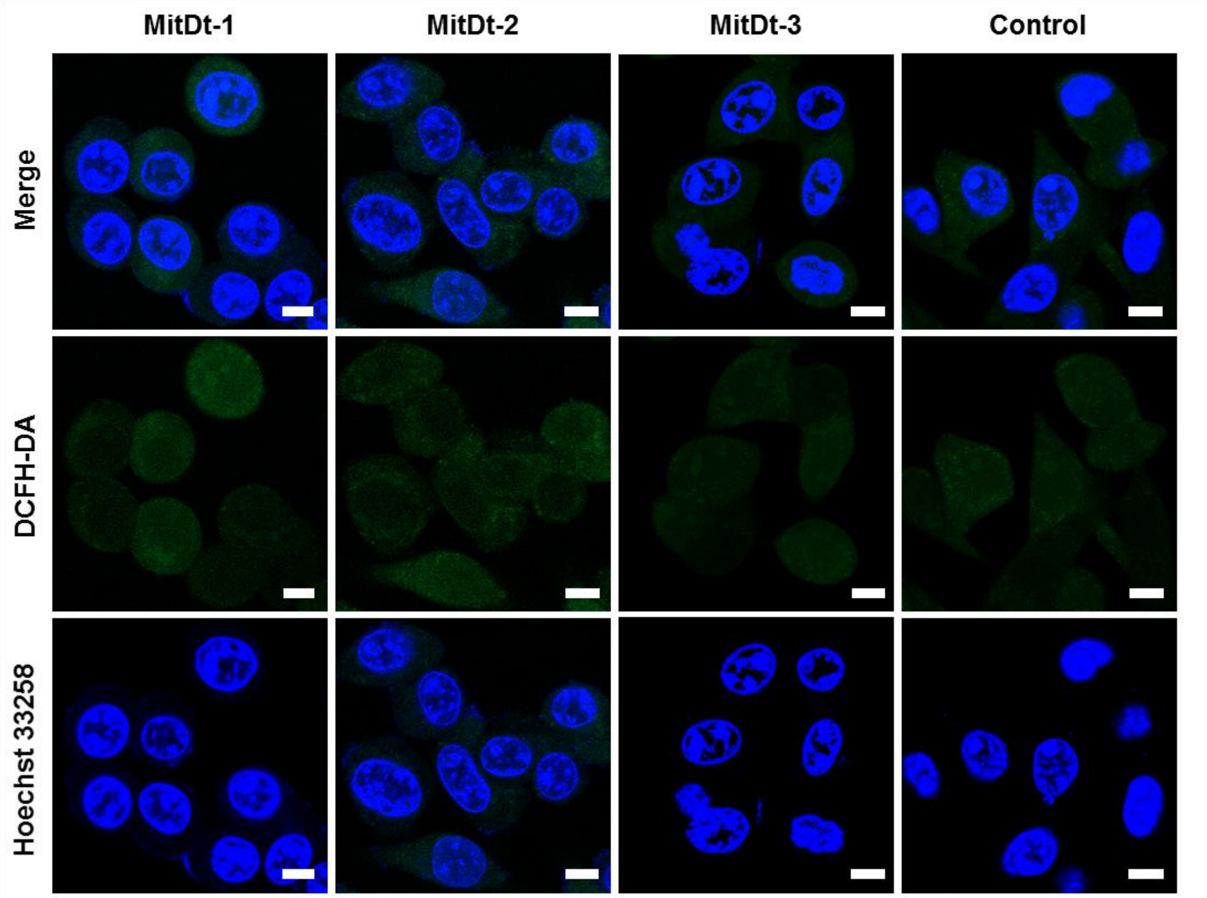

Figure S6. Visualization of in vitro ROS production of brominated MitDt group (10 μM) using DCFH-DA without laser in MCF-7, measured by confocal laser scanning microscopy. (Scale bar = 10 μm)

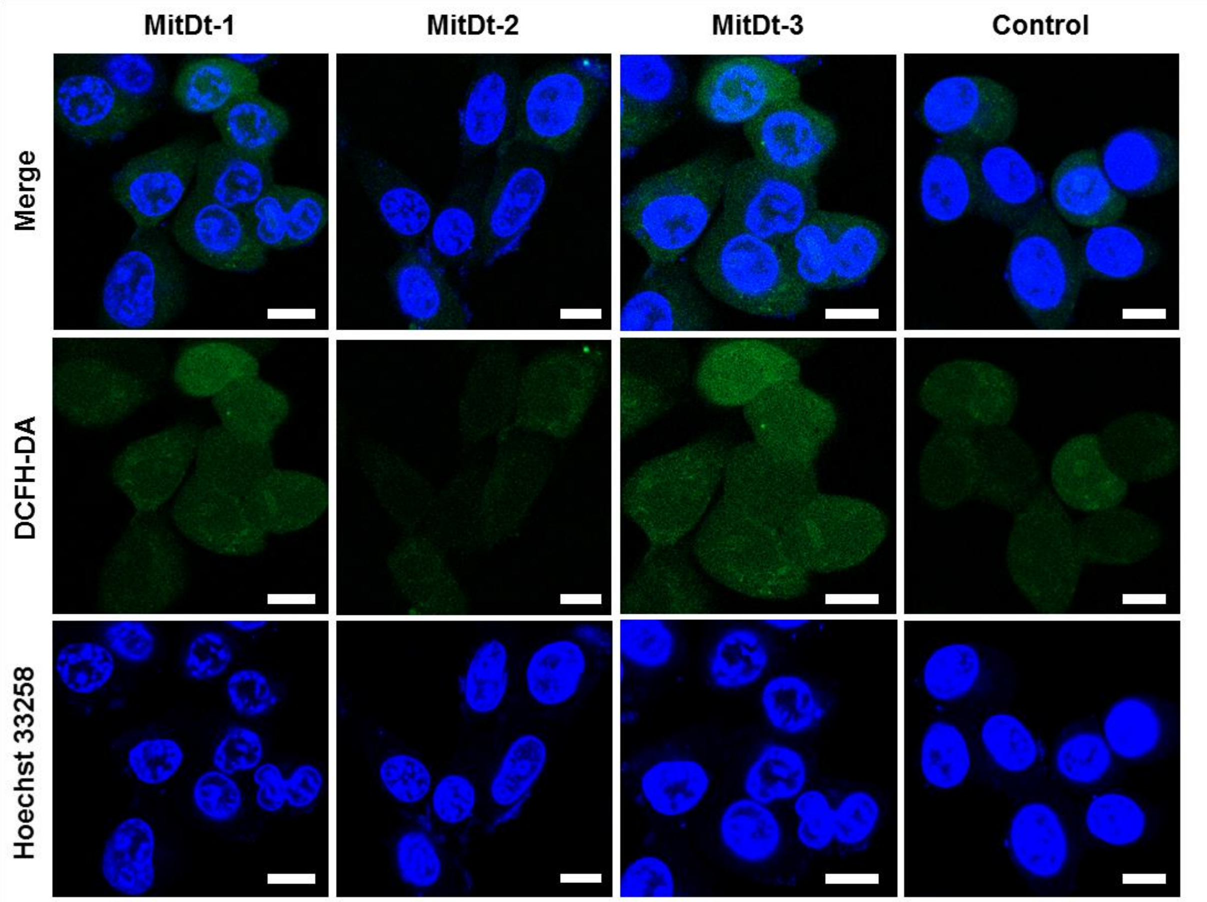

Figure S7. Visualization of in vitro ROS production of brominated MitDt group (10 μM) using DCFH-DA after 5 min NIR laser irradiation (100 mW/cm^2) in MCF-7, measured by confocal laser scanning microscopy. (Scale bar = 10 μm)

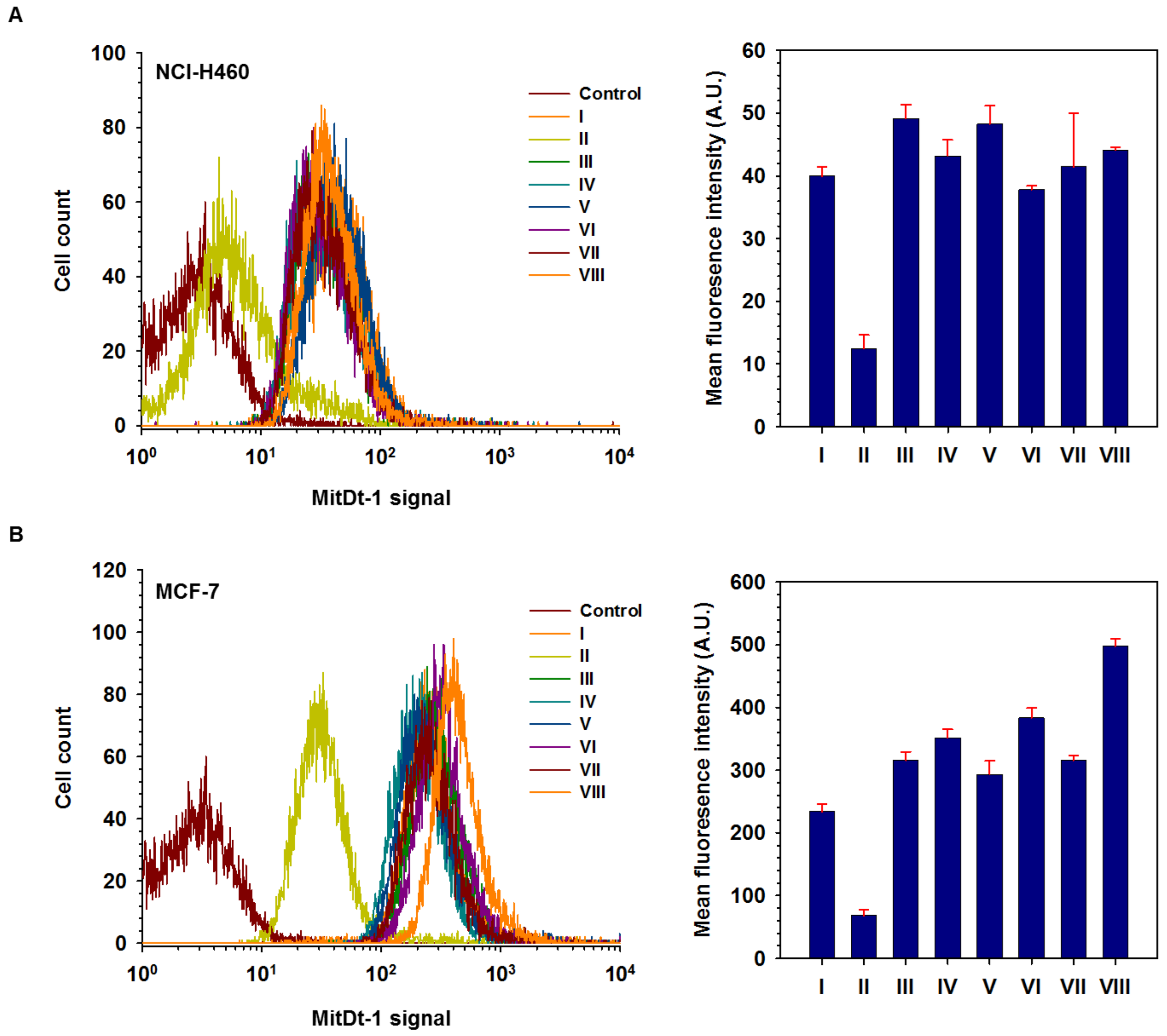

Figure S8. Cellular uptake mechanism of MitDt-1 in NCI-H460 (A) and MCF-7 (B) Cell lines determined using flow cytometry after pre-treatment with various agents: I) 37 °C, II) 4 °C, III) sulfobromophthalein (BSP, 250 mM, for 5 min), IV) 2-Deoxy-D-glucose (2-DG, (150 × 10^{-3} M, 45 min), V) Chlorpromazine (CPZ, 30 µM, 1 h), VI) 5-(N-ethyl-N-isopropyl)-amiloride (EIPA, 20 µM, 1 h), VII) Hypertonic sucrose (HS, 0.4 M, 1 h), and VIII) Methyl-β-cyclodextrin (MβCD, 10 mM, 1 h).

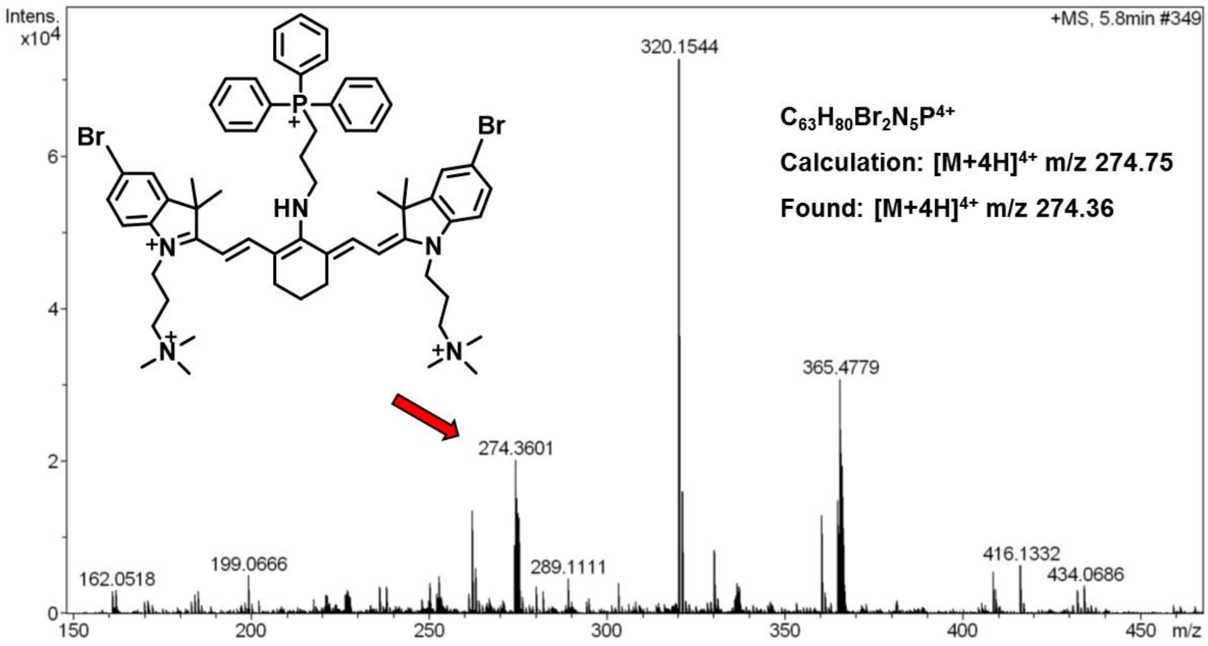

Figure S9. HR-MS of MitDt-1.

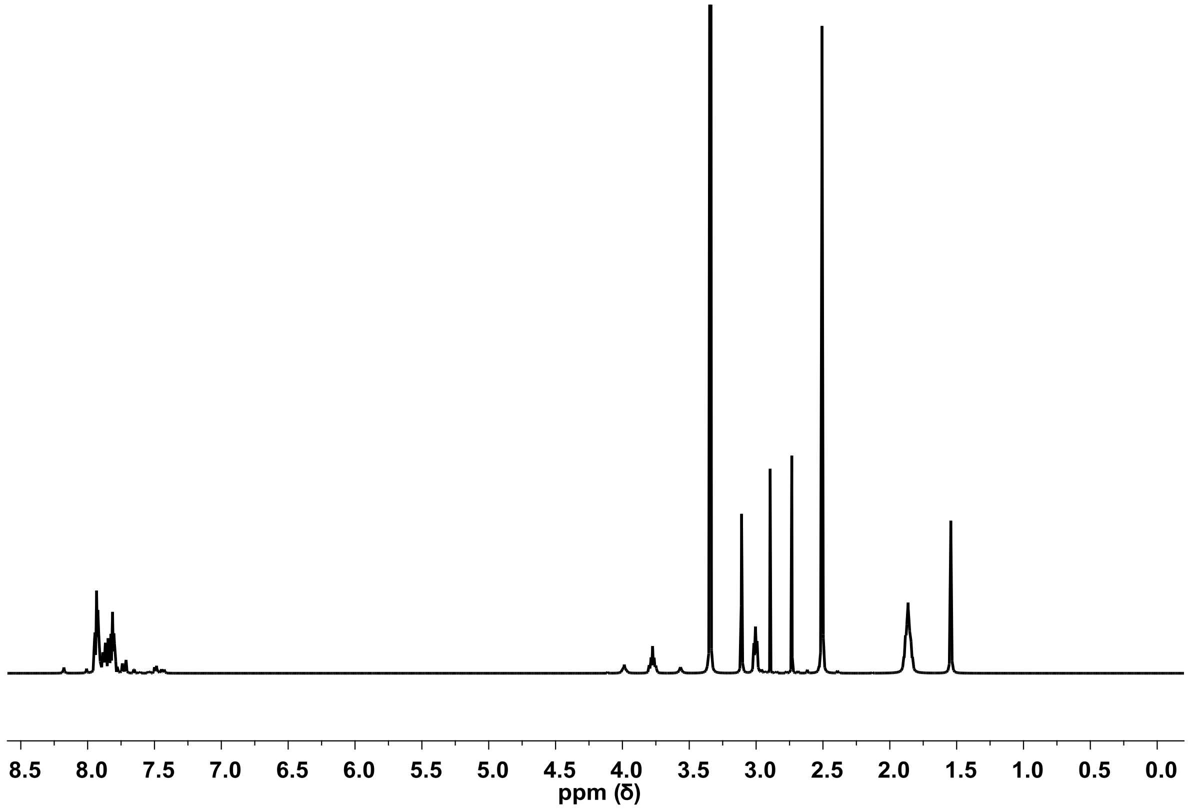

Figure S10. ^{1}H NMR of MitDt-1.

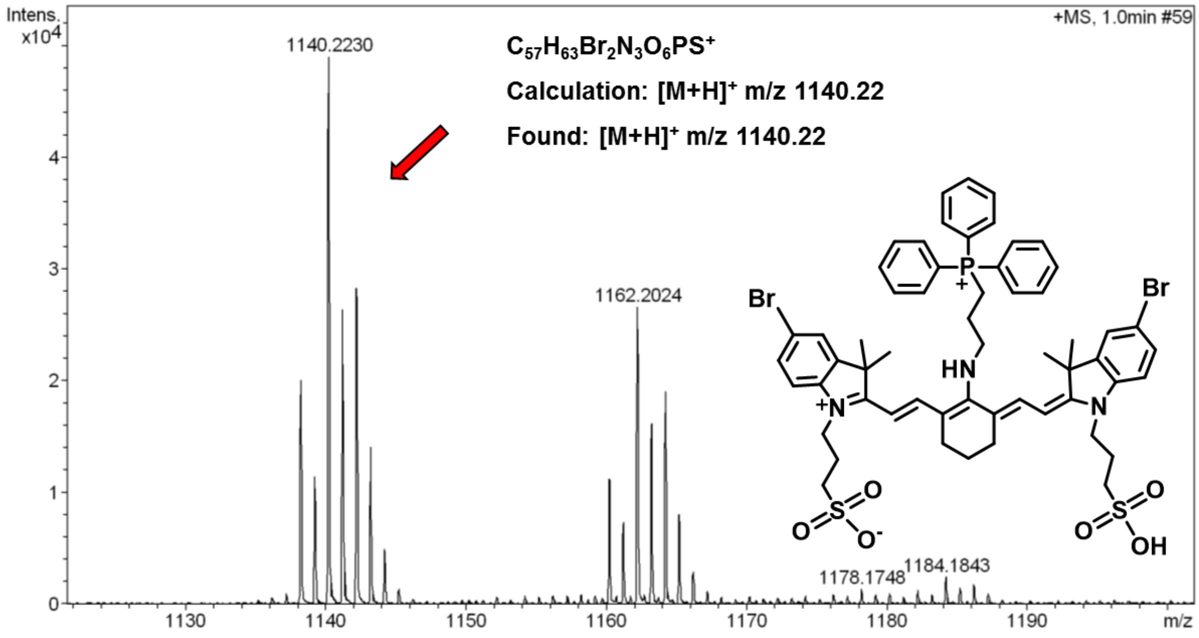

Figure S11. HR-MS of MitDt-2.

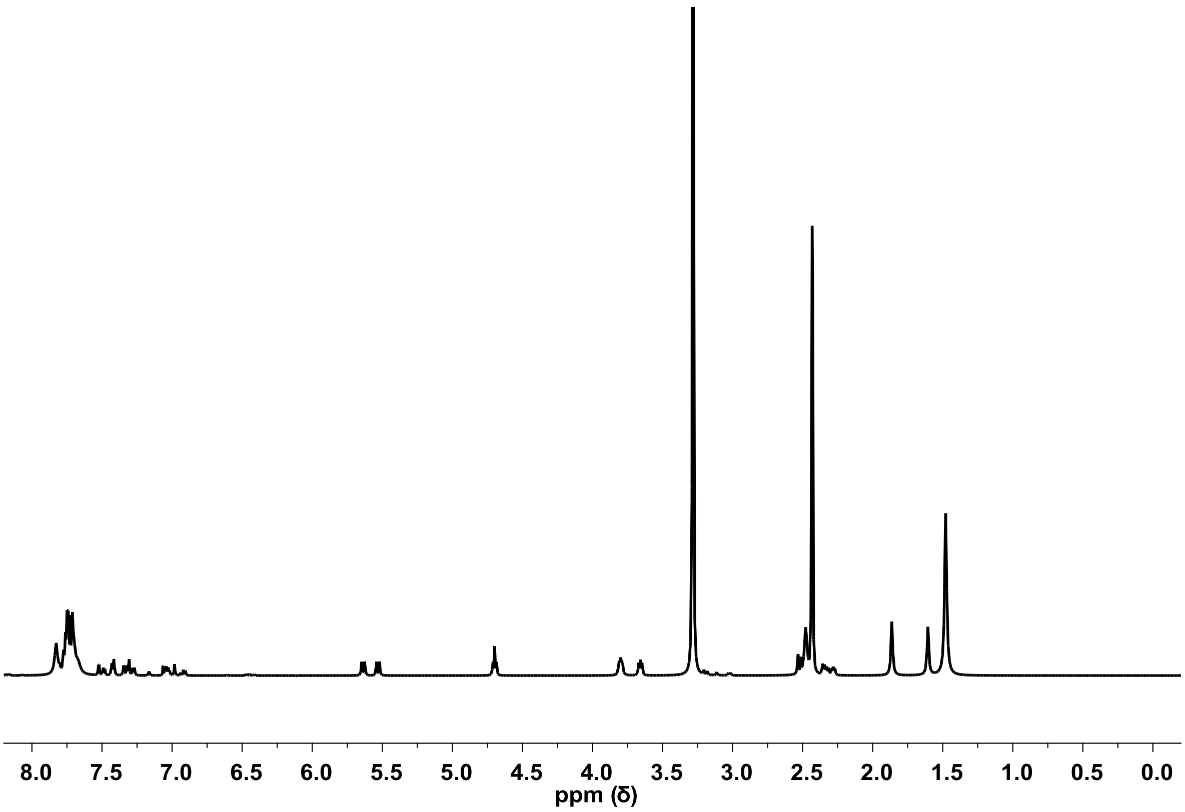

Figure S12. ^{1}H NMR of MitDt-2.

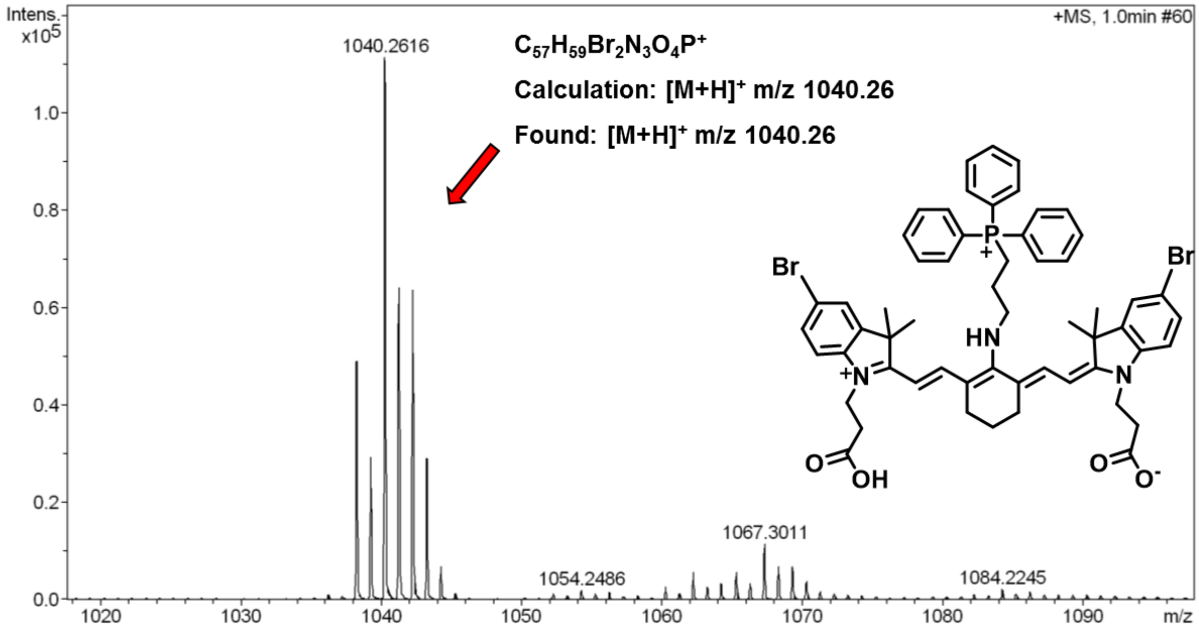

Figure S13. HR-MS of MitDt-3.

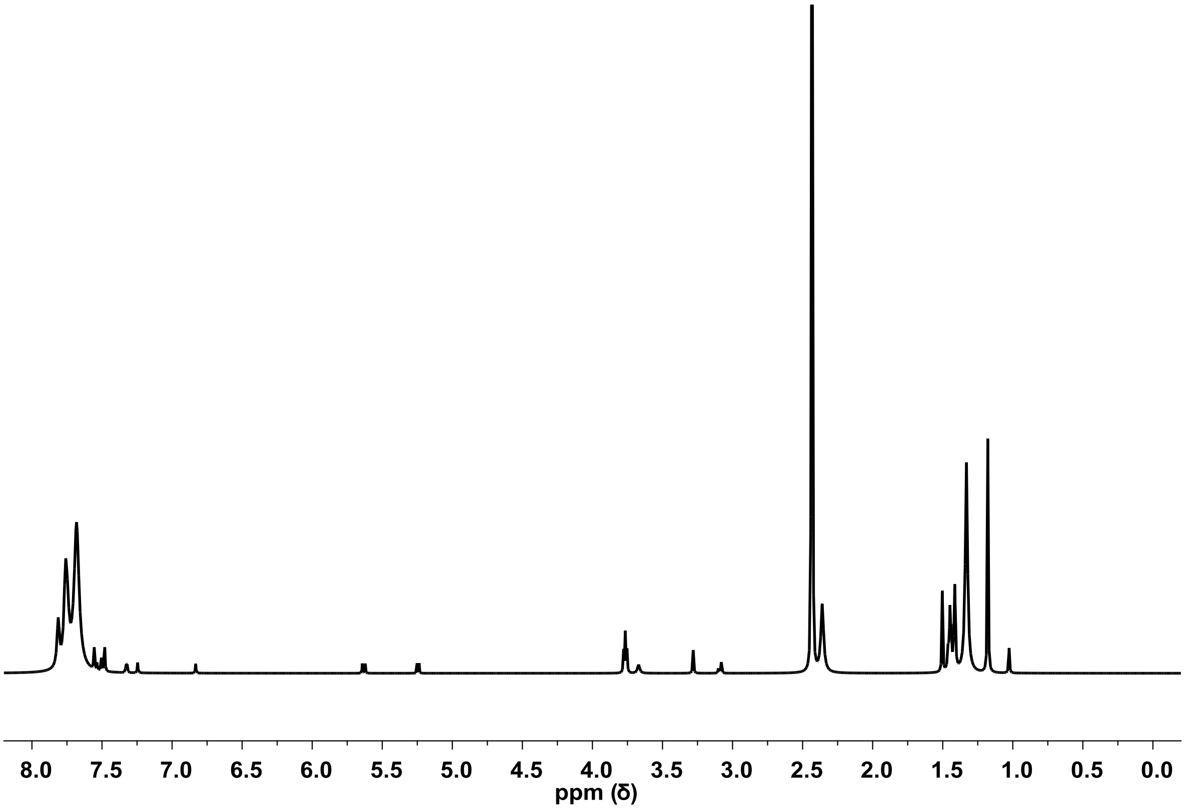

Figure S14. ^{1}H NMR of MitDt-3.

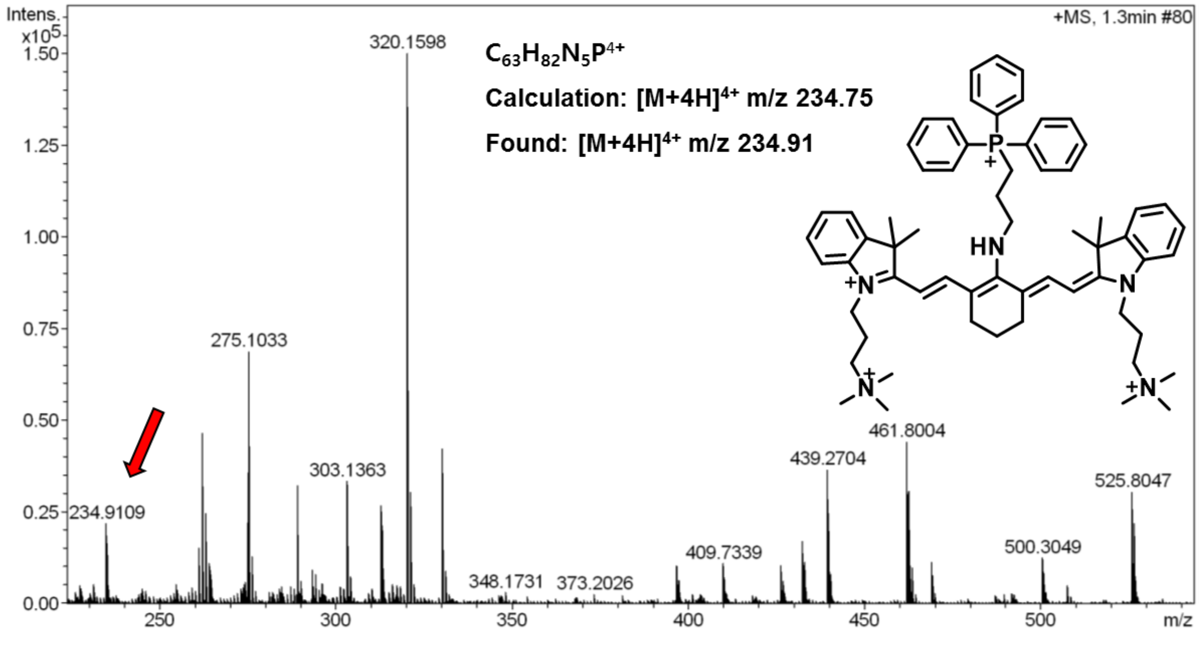

Figure S15. HR-MS of MitDt-4.

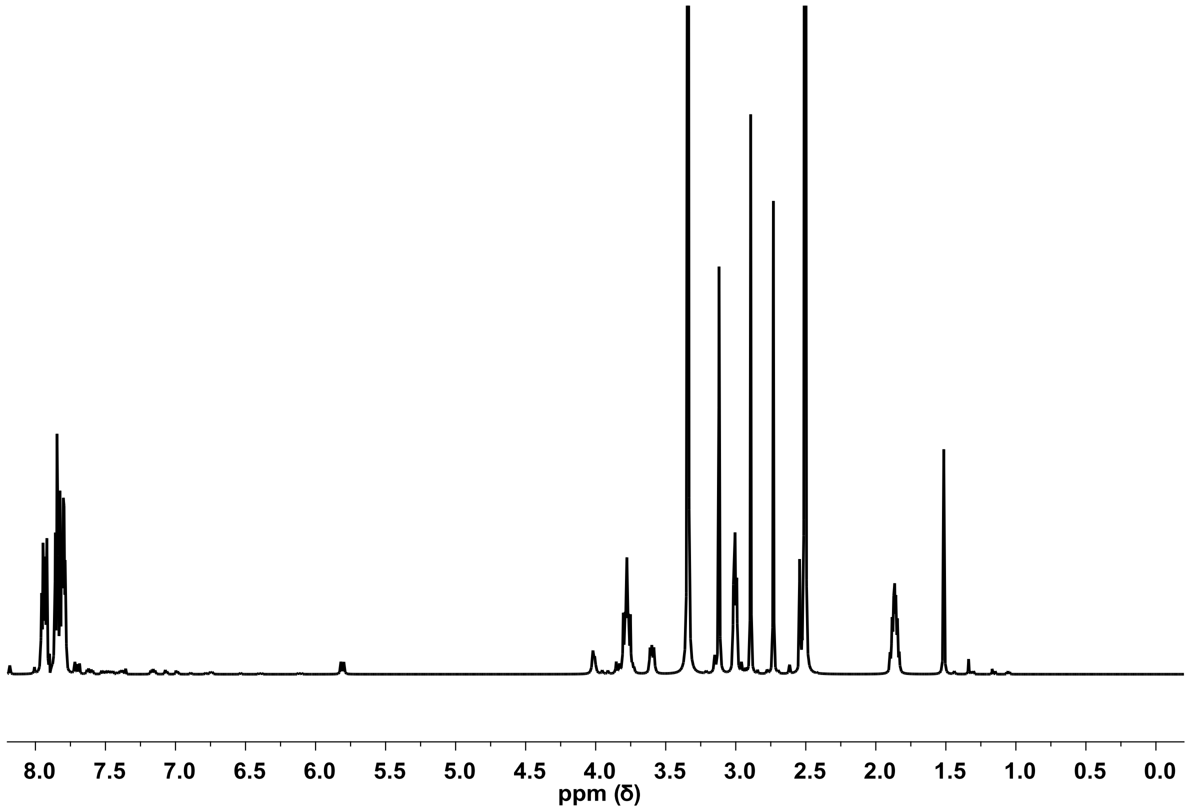

Figure S16. ^{1}H NMR of MitDt-4.

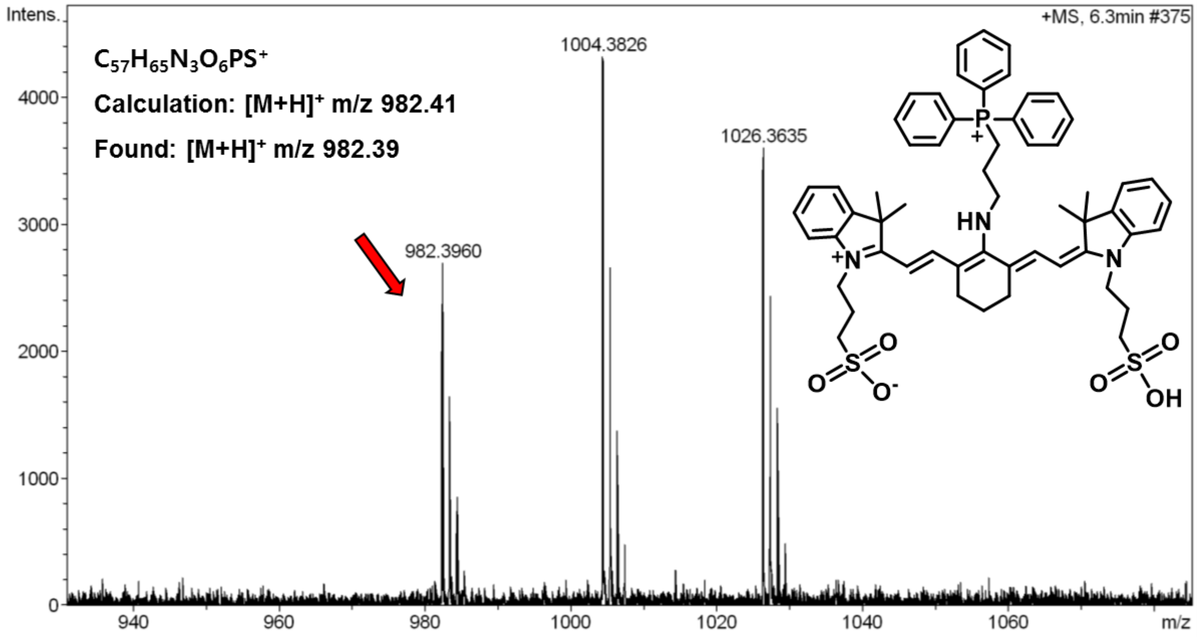

Figure S17. HR-MS of MitDt-5.

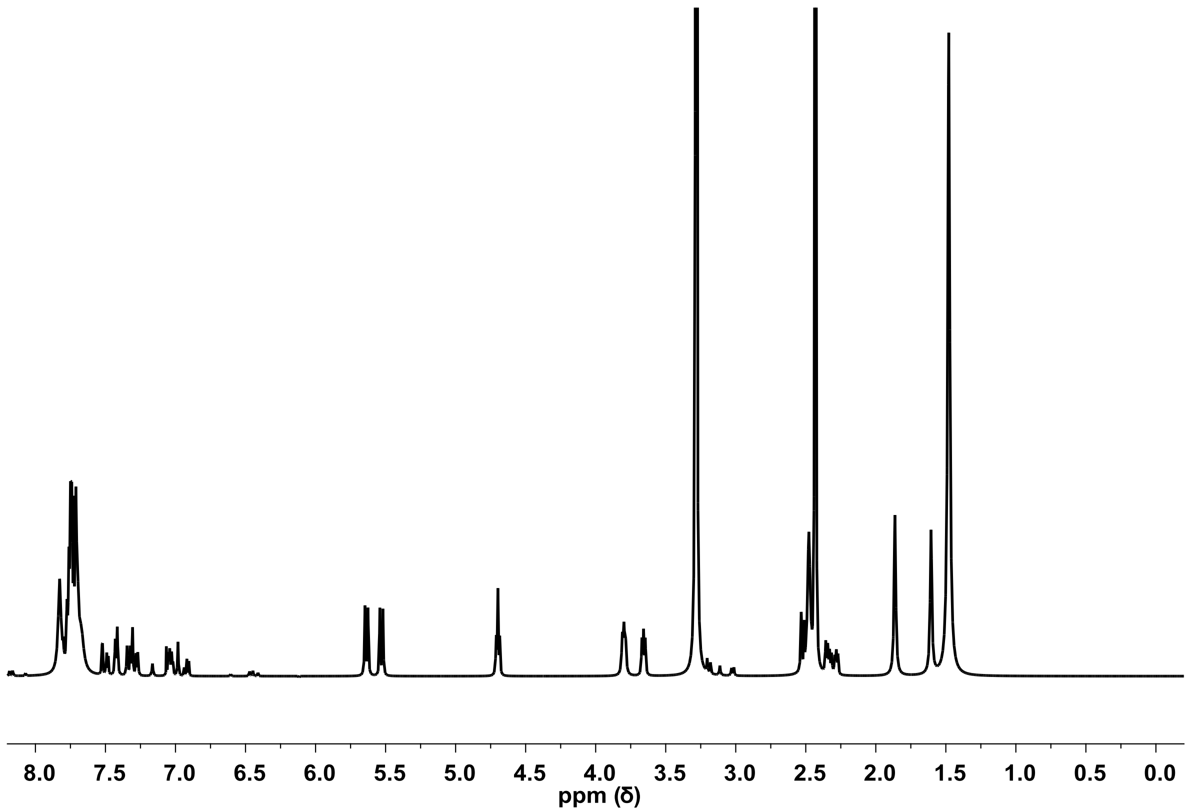

Figure S18. ^{1}H NMR of MitDt-5.

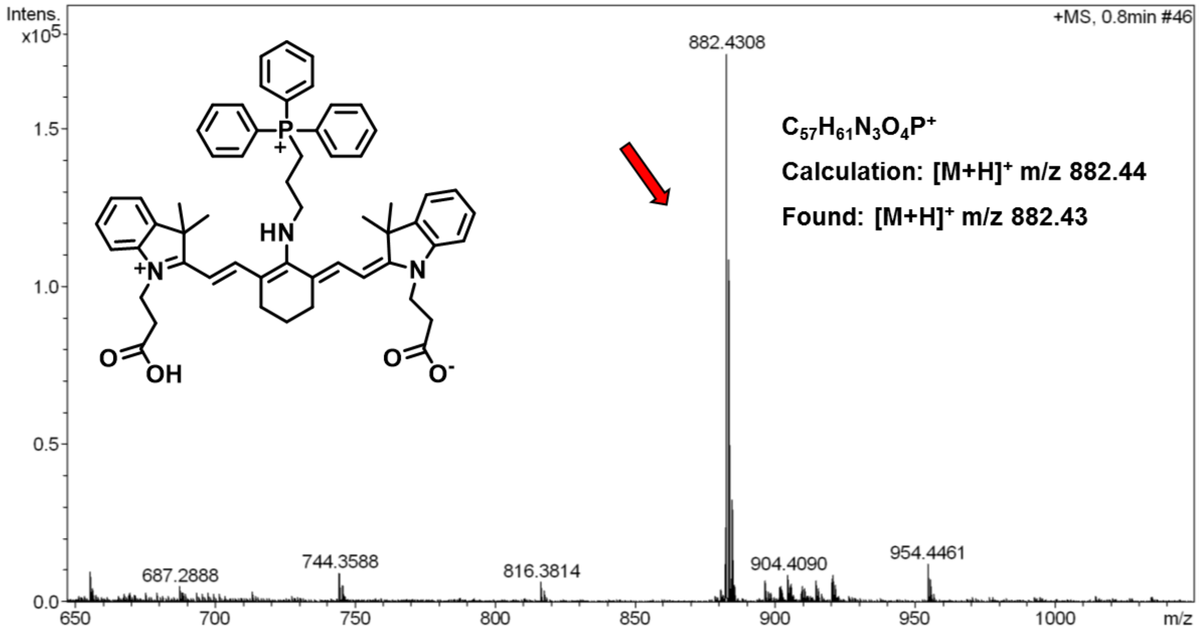

Figure S19. HR-MS of MitDt-6.

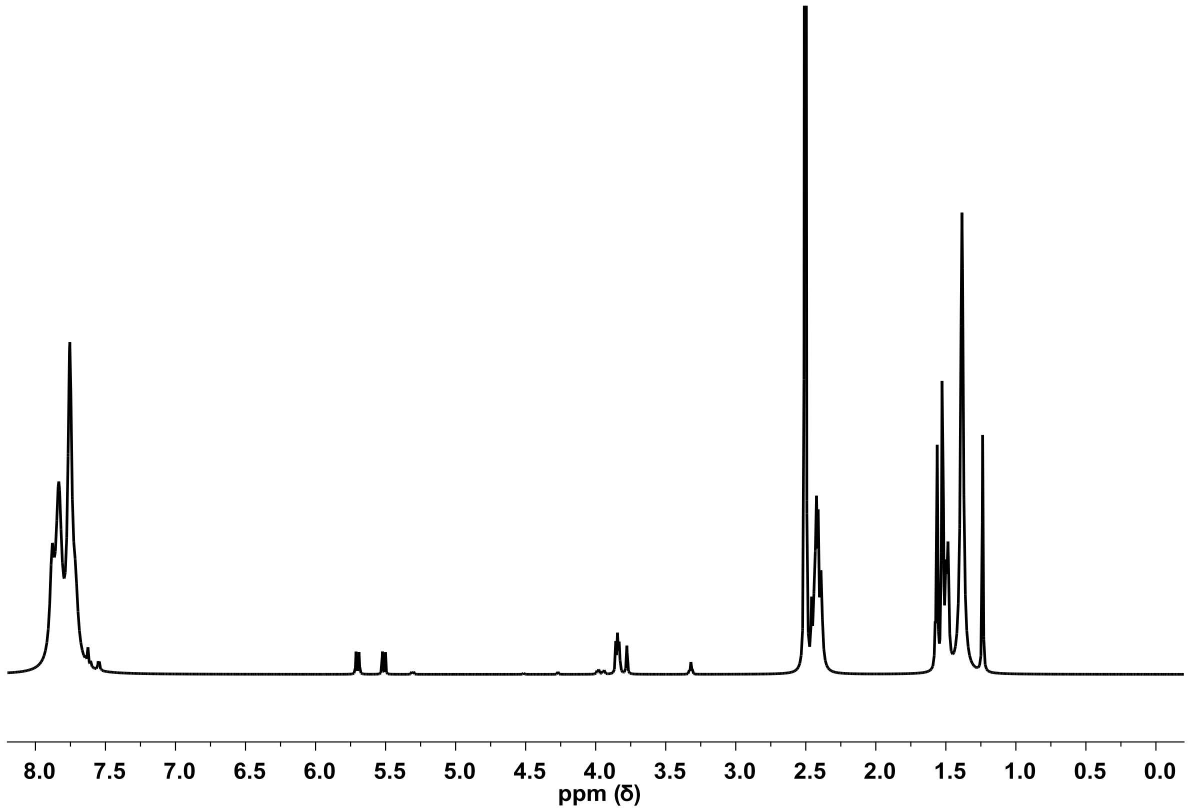

Figure S20. ^{1}H NMR of MitDt-6.

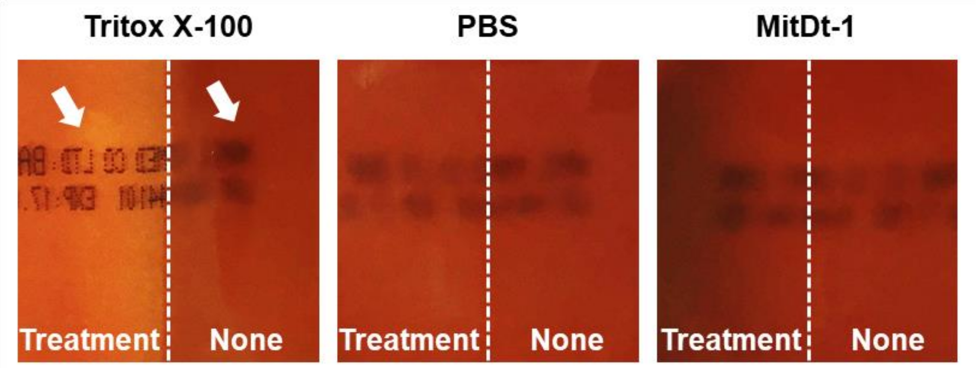

Figure S21. Blood compatibility confirmation. The hemolytic activity assay was conducted using 5% horse blood agar plates treated with triton X-100, PBS, and MitDt-1 (100 μM, 200 μl in PBS). Triton X-100 was used as a negative control.

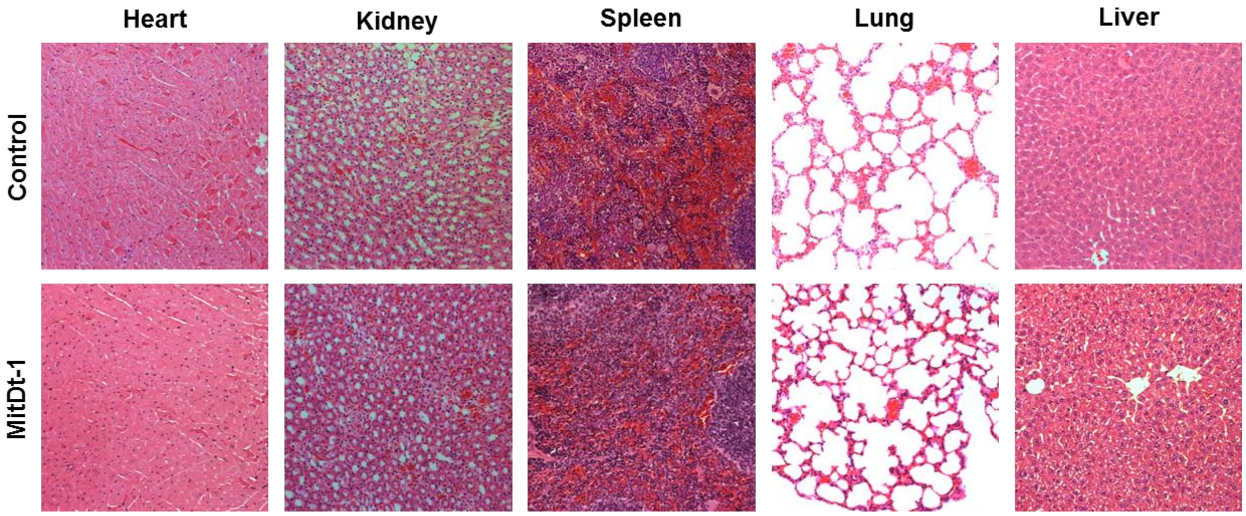

Figure S22. H&E staining images of extracted organs (heart, kidneys, spleen, lungs, and liver) treated with 100 μM of MitDt-1 (200 μl in PBS) after 24 h of injection (200x).

References

[1] a) A. Punzi, M. Capozzi, V. Fino, C. Carlucci, M. Suriano, E. Mesto, E. Schingaro, E. Orgiu, S. Bonacchi, T. Leydecker, *J. Mater. Chem. C* **2016**, *4*, 3138; b) E. A. Owens, H. Hyun, S. H. Kim, J. H. Lee, G. Park, Y. Ashitate, J. Choi, G. H. Hong, S. Alyabyev, S. J. Lee, *Biomed. Mater.* **2013**, *8*, 014109.

[2] a) H. Hyun, M. W. Bordo, K. Nasr, D. Feith, J. H. Lee, S. H. Kim, Y. Ashitate, L. A. Moffitt, M. Rosenberg, M. Henary, *Contrast Media Mol. Imaging* **2012**, *7*, 516.

[3] A. Kaur, K. W. L. Brigden, T. F. Cashman, S. T. Fraser, E. J. New, *Org. Biomol. Chem.* **2015**, *13*, 6686.
